# Beyond reweighting: On the predictive role of covariate shift in effect generalization

**DOI:** 10.1073/pnas.2427181122

**Published:** 2025-11-03

**Authors:** Ying Jin, Naoki Egami, Dominik Rothenhäusler

**Affiliations:** ^a^Data Science Initiative & Department of Health Care Policy, Harvard University, Cambridge, MA 02138; ^b^Department of Political Science, Columbia University, New York, NY 10027; ^c^Department of Statistics, Stanford University, Stanford, CA 94305

**Keywords:** generalizability, external validity, distribution shift, replication studies

## Abstract

Many existing approaches to generalizing statistical inference amid distribution shift operate under the covariate shift assumption, which posits that the conditional distribution of unobserved variables given observable ones is invariant across populations. However, recent empirical investigations have demonstrated that adjusting for shifts in observed variables (covariate shift) is often insufficient for generalization. In other words, covariate shift does not typically “explain away” the distribution shift between populations. As such, addressing the unknown yet nonnegligible shift in the unobserved variables given observed ones (conditional shift) is crucial for generalizable inference. In this paper, we present empirical evidence from two large-scale multisite replication studies indicating that covariate shift can help predict the strength of unknown conditional shift. Analyzing 680 studies across 65 sites, we find that even though the conditional shift is nonnegligible, its strength can often be bounded by that of the observable covariate shift. This pattern only emerges when the two sources of shifts are quantified by our proposed standardized, pivotal measures. We then interpret this phenomenon by connecting it to similar patterns that can be theoretically derived from a random distribution shift model. Finally, we demonstrate that exploiting the predictive role of covariate shift leads to reliable and efficient uncertainty quantification for target estimates in generalization tasks with partially observed data. Overall, our empirical and theoretical analyses highlight an alternative perspective on the problem of distributional shift, generalizability, and external validity.

## Significance

Generalizing scientific findings across diverse populations is fundamental to many scientific fields and policymakers who use such analyses to guide decision-making. Traditional methods often account for the differences in populations by reweighting observed covariates, assuming only observed variables shift across the populations. Analyzing large-scale replication studies in the social sciences, we empirically demonstrate that i) shifts in unobserved variables are common, but ii) such shifts can be predicted from shifts in observed covariates. We propose a statistical theory of distributional shifts to explain this predictive, rather than merely explanatory, role of covariates in effect generalization. Our results serve as the empirical and conceptual basis for developing new statistical methods for generalizability and external validity.

Distribution shift is a central issue in generalizing statistical evidence from an observed (source) population to a new, at most partially observed (target) population, with significant implications in many domains. For instance, in the medical and social sciences, researchers/policymakers seek to leverage existing randomized control trials to estimate the treatment effect on a new cohort to guide clinical decisions or policy making (1–7). However, the challenge lies in whether statistical methods can capture the changes between populations to produce credible predictions of target effects.

To address the generalizability question, many statistical methods operate under assumptions positing that observed variables capture all distributional differences between populations. These assumptions can often be described as covariate shift, that is, the distribution of covariates observed in both populations can change, while the conditional distribution of the outcomes (unobserved in the target population) given the observed covariates remains invariant. For example, the distribution of age, gender, and education can differ across populations (e.g., due to convenience sampling), but the conditional treatment effect is the same for individuals with the same covariate profiles. Under this common assumption, adjusting for shift in the observed covariates, either by reweighting based on density ratios or estimating the heterogeneous covariate–outcome relationship (8–12), is sufficient for unbiased estimation of the target parameters. This common approach highlights the role of covariate shift in explaining away the distribution shift.

Given its popularity, a series of recent papers (13–15) have empirically evaluated the performance of generalization estimators based on the covariate shift assumption by comparing them against experimental benchmark estimates. Although each paper focuses on different domains, a common yet somewhat surprising finding is that observed covariate shift often can only explain a small proportion of the distributional shift in real-world applications. This implies two pessimistic messages: 1) adjusting for observed covariate shift may be insufficient for generalization, and 2) the remaining, unobserved conditional shift (i.e., shift in the conditional distribution of the outcomes given the observed covariates) is “larger” than the observed covariate shift. As such, it remains unclear how the conditional shift may be addressed for effect generalization in practice even in well-controlled settings.

## 1.1. This Work: The Predictive Role of Covariate Shift.

**Fig. 1.**
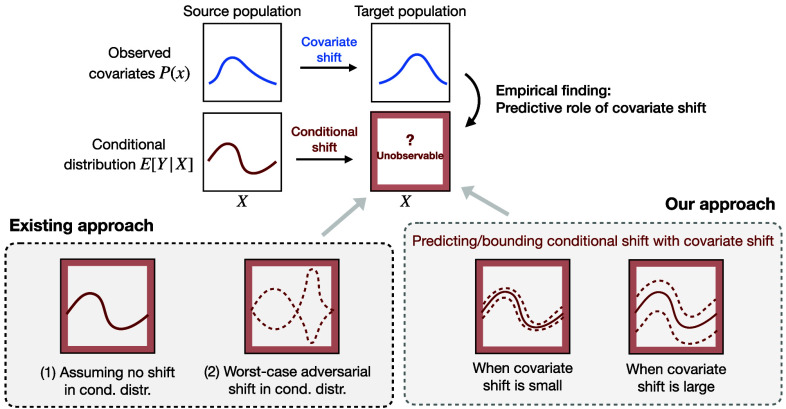
Overview of the problem and our approach. Effect generalization from source and target populations needs to address the distribution shift consisting of the observed covariate shift and unobserved conditional shift. We argue a predictive role of covariate shift in bounding the strength of unknown conditional shift, which is supported by our empirical findings and leads to reliable and efficient generalization.

In this paper, we introduce a different role of covariate shift in predicting the unknown shift in the conditional distribution for generalization (Fig. 1). The distribution shift between the source and target populations consists of the observed covariate shift and unobserved conditional shift, the latter being a key challenge in a generalization task. In contrast to existing approaches that either i) assume no conditional shift, or ii) establish worst-case bounds based on adversarial shift in the conditional distribution, we argue that the strength of covariate shift can bound that of the unknown conditional shift. Exploiting this bounding relationship is useful in effect generalization with improved validity and efficiency.

**Fig. 2.**
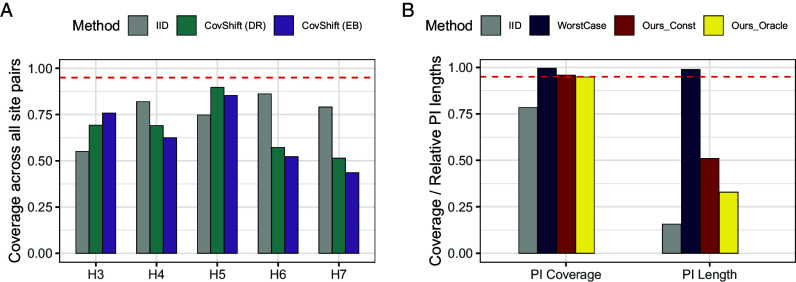
Preview of results. Panel (*A*): Insufficient explanatory role of covariate shift: Empirical coverage of prediction intervals based on i.i.d. assumption (grey) and covariate shift assumption (green and purple), showing covariate shift cannot explain away distribution shift across sites. Panel (*B*): Reliable and efficient effect generalization based on the predictive role of covariate shift: Empirical coverage of prediction intervals based on i.i.d. assumption (grey), worst-case bounds (dark blue), and our method with the belief that conditional shift is bounded by covariate shift (red) or with knowledge of their relative strength (yellow).

Our proposal is supported by empirical evidence from two well-known, large-scale multisite replication projects—the Pipeline project (16) and the Many Labs 1 project (17)—from the social sciences, analyzing a total of 680 studies across 65 sites examining 25 hypotheses.^*^ To ensure faithful evaluation, since we have no access to the underlying population parameters, we build prediction intervals—based on various distribution shift assumptions—for estimators in target populations (including our proposed ones built upon empirical findings) and use their empirical coverage to examine the plausibility of the assumptions they are based upon. Fig. 2 previews our empirical results.

We begin by examining common approaches that either ignore distribution shift or assume covariate shift (Section 2.3). In the two replication projects, the explanatory role of covariate shift is limited as evident from the low coverage of prediction intervals, complementing existing work that either examine pairs of studies (14) or mean squared errors (15, 18). As shown in Panel (*A*) of Fig. 2, even for controlled multisite replication studies, distribution shifts across sites are not negligible (methods that assume no distributional shift (IID) do not achieve valid coverage). Furthermore, observed covariate shift cannot explain away the total distributional shift, as methods that only adjust for observed covariate shift (CovShift) do not achieve valid coverage, either.

We then proceed to compare the strengths of the observed covariate shift and the conditional shift (Section 3.2). In stark contrast with the pessimistic conjectures in previous works, we find that conditional shift is often smaller than covariate shift across different applications and comparisons. However, this empirical pattern became clear only after we measured covariate and conditional shifts with proper standardization.

We interpret our empirical findings by connecting them to similar patterns that can be theoretically derived under a recently proposed random distribution shift model (19–21) (Section 3.3). Under this model, one expects to observe smaller conditional shift than covariate shift when the probability space is randomly perturbed in a way that does not favor any direction yet some component of the observed data, which is the treatment assignment here, is kept invariant. This model describes scenarios where the difference between the source and target distributions is not adversarial but is contributed by many small and random factors. Such scenarios are common in collaborative replication studies and potentially other carefully controlled studies where replicators try their best to mimic the original study design and population, but they have to deviate due to logistical and other constraints.

Finally, we demonstrate the effectiveness of exploiting this predictive role in effect generalization, again (for evaluation purposes) by examining the empirical coverage of prediction intervals that aim to address the unknown conditional shift (Section 4). Panel (*B*) of Fig. 2 previews key takeaway messages. Prediction intervals derived from this framework maintain valid coverage while yielding substantially shorter intervals. This reveals that the predictive role is stable across contexts and permits effective empirical calibration. In contrast, existing methods assuming worst-case conditional shift (WorstCase) achieve valid coverage when the worst-case shift strength is (unrealistically) calibrated by data, but at the expense of too wide intervals.

Overall, our empirical and theoretical analyses suggest a different way to approach the problem of distributional shift, generalizability, and external validity. Most existing methods either i) assume no shift in the unobserved conditional shift or ii) assume shift in the unobserved conditional shift is bounded, and search for the worst-case scenarios that tend to be extremely adversarial. Instead, we offer a data-adaptive middle ground—shift in the unobserved conditional shift is nonnegligible but is predictable from the observed covariate shift. Our results shall serve as the empirical and conceptual basis for developing new methods and models beyond the covariate shift assumption.

## 1.2. Scope of the Paper.

We emphasize that the main objective of this paper is to offer empirical and theoretical evidence supporting a particular perspective on real-world distributional shifts. The random distribution shift modeling assumption offers a perspective to justify our empirical findings, yet we do not anticipate it to be universally grounded. In particular, we limit the interpretation of our results to contexts similar to multisite replication studies where data are collected in a “natural” manner, with distribution shifts arising from random, unintended factors while the experimenters try to maintain consistency. In other words, the two projects provide a testbed for distribution shifts that emerge due to inevitable deviations despite well-controlled experimental settings (22–24). Counterexamples include studies where researchers purposively change the recruitment criterion across sites, e.g., one site deliberately focuses on university students whereas another site recruits only middle-aged participants, or when researchers purposively change experiment procedures, e.g., the experiment materials in one study are intentionally modified (we discuss robustness analyses in Section 5).

We also note that our evaluation mainly focuses on uncertainty quantification, that is, whether statistical methods can produce reliable prediction intervals for the actual estimates from data in the target population. Focusing on prediction intervals is inevitable since the underlying parameter is not accessible for evaluation purposes. In addition, uncertainty quantification offers a more comprehensive assessment than evaluating the consistency or unbiasedness of point estimates (see Section 1.3 for more discussion).

## 1.3. Related Work.

### 1.3.1. Reweighting in causal inference.

Using reweighting to generalize from one population to another population has a long history in causal inference. Early examples include Horvitz-Thompson (25) and Hájek’s estimator. Inverse probability weights are often unstable in practice. This has spurred the development of procedures that use outcome models to reduce variance (26) and balancing weight procedures that penalize the weights (27, 28). Modern reweighting procedures were used to generalize the results of experiments from one site to another (e.g., refs. 4, 5, 8, 11, and 29–32). See refs. 33 and 34 for recent reviews.

### 1.3.2. Empirical evaluation of generalization.

This work adds to several recent works empirically evaluating generalization procedures that use unit-level data to generalize from one site to another. Ref. 13 diagnose how much of the drop of prediction performance can be attributed to covariate shift vs. concept Y|X shift. Refs. 14 and 15 investigate how much of the discrepancy between causal effect estimates in different sites is due to unit-level covariates, among other factors. In welfare-to-work experiments, ref. 15 found that less than 10% of discrepancies between sites is explained by changes in covariate distributions. This work echoes these works on the insufficient explanatory role of covariate shift. An important distinction is that our evaluation leverages the coverage of prediction intervals over many replication studies, which offers more comprehensive and faithful evaluation than methods that evaluate one pair of studies for a hypothesis (13, 14) or examine the mean squared errors (15, 18). For example, while (18) find in another multisite replication dataset that covariate adjustment leads to *unbiased* estimators (with bias averaged over multiple sites) for target estimates, it may still underestimate the variability if the conditional shift leads to discrepancies that are mean zero when averaged over studies but have nonnegligible magnitude. More importantly, we also investigate a predictive role of covariate shift that can inform reliable generalization in practice.

### 1.3.3. Heterogeneity and meta-analysis in replicability.

Multisite replication projects have been used to examine the heterogeneity in effect estimates across sites (35–39). A prominent distinction is that these works often measure certain global notions of heterogeneity via meta-analysis (40), while we focus on generalization from one site to another. Methodologically, our generalization methods are applicable when data from only the source and target sites are available, whereas meta-analysis needs data from many sites. In addition, these works provide echoing messages for weak explanatory roles of observed factors (35, 38) or complementary messages for design and estimation uncertainty (39, 41); the latter may be interpreted as “random” shifts if not documented.

### 1.3.4. Covariate and conditional shift in machine learning.

The term covariate shift was first introduced by ref. 42 and has become one of the standard domain adaptation models; see refs. 43 and 44. Most commonly, covariate shift is addressed via importance weighting with the density ratio, which can be estimated directly, e.g., via a classifier (45). Similarly, density ratio reweighting is a standard approach to addressing covariate shift for statistical estimation and inference. The conditional shift we study is related to the notion of concept drift in machine learning (46, 47). The techniques for addressing these shifts in prediction problems serve distinct goals than our estimation and inference problems.

## 2. Motivating Applications and Methodological Problem

We introduce our motivating applications and illustrate the core methodological challenges in generalization.

### 2.1. Motivating Applications: Multisite Replication Projects.

In this paper, we use two large-scale multisite replication projects from the social sciences to empirically investigate the role of covariate shifts in generalization. The Many Labs 1 project (17) evaluates the replicability of 13 classic and contemporary experimental findings in the social sciences, ranging from gain vs. loss framing (48) to sex differences in implicit attitudes toward math (49), across 36 independent data collection sites. Similarly, in the Pipeline project (16), 25 laboratories across the world (contributing 29 populations) independently replicate experiments for 10 scientific hypotheses concerning moral judgment, which is a well-known theory in psychology. Combining the two replication projects, we analyze 680 studies across 65 sites, examining 25 research hypotheses. This scale allows us to assess the proposed role of covariate shifts across diverse empirical settings.

Several features of these multisite replication projects make them suitable for evaluating distribution shifts in generalization. First, we can mimic the real-world generalization task by generalizing an effect estimate from one source site to another target site. Unlike the real generalization task, we have access to the effect estimate from the target site, and therefore, we can empirically evaluate the performance of common generalization estimators based on the covariate shift assumption and our proposed estimator, without simulating data from the artificial data-generating process. Second, in these replication projects, multiple laboratories follow the same experimental process as much as they can, known as direct replications. As a result, the measurement of the outcome variable and treatment variable is consistent across sites, and the interpretation of the covariate shift and the unobserved conditional shift becomes clearer. Finally, the two replication projects differ in how laboratories are recruited. In the Pipeline project (16), laboratories are invited by the project lead because they had “access to a subject population in which the original finding was theoretically expected to replicate using the original materials” (p. 57). Therefore, sites were selected such that distributional shifts between them are expected to be small, or at least without intentional manipulation and adversarial changes. On the other hand, in the Many Labs 1 project (17), laboratories voluntarily participated in the project without specific eligibility criteria related to whether each site was expected to replicate the original finding. Here, sites were selected conveniently but again “naturally” without explicit intention and manipulation. This variation in site selection enables us to empirically evaluate distributional shifts in diverse scenarios.

The datasets are processed based on the raw data and scripts published by the original authors. In both projects, the covariates include demographic variables such as political ideology, gender, age, education, and income. See *SI Appendix*, section 1 for details about the datasets and preprocessing.

### 2.2. Notation and Setup.

To formally discuss the generalization problem, we introduce some notation. While we tailor our notation to the two projects above for concrete presentation, the same general framework can be applied to any generalization setting across sites.

We first index the hypotheses by k∈{1,⋯,K} and the sites by j∈{1,⋯,N}. Each hypothesis k is tested by a randomized experiment in a subset of sites $J_{k}\subseteq \left\{1,\cdots ,N\right\}$, following the same experimental protocol. Each site $j\in J_{k}$ independently collects $n_{j}^{\left(k\right)}\in N$ participants and collects data $D_{j}^{\left(k\right)}={\left\{X_{i}^{\left(j,k\right)},T_{i}^{\left(j,k\right)},Y_{i}^{\left(j,k\right)}\right\}}_{i=1}^{n_{j}^{\left(k\right)}}$, where $X_{i}$ is the covariates, $T_{i}\in \left\{0,1\right\}$ is the binary treatment, and $Y_{i}$ is the outcome(s). Then, within each site, we can define the parameter of interest $\theta_{j}^{\left(k\right)}$ and its consistent and asymptotically normal estimator ${\hat{\theta }}_{j}^{\left(k\right)}$, which is a function of $D_{j}^{\left(k\right)}$. In our applications, most of them consider the average treatment effect (ATE) as $\theta_{j}^{\left(k\right)}$ and use a t test that compares the sample mean of treated and control groups as ${\hat{\theta }}_{j}^{\left(k\right)}$. Some hypotheses are tested with $\theta_{j}^{\left(k\right)}$ being the mean of outcomes and ${\hat{\theta }}_{j}^{\left(k\right)}$ being a paired t test comparing two outcomes. The specific hypotheses and tests are summarized in *SI Appendix*, Tables S2 and S4.

We assume $D_{j}^{\left(k\right)}$ are drawn i.i.d. from an underlying (hypothetical) superpopulation $S_{j}^{\left(k\right)}$, and datasets are independent across sites $j\in J_{k}$ for each hypothesis k. Importantly, the underlying data generating process $S_{j}^{\left(k\right)}$ may vary across sites $j\in J_{k}$ since there might exist distribution shifts.

We consider the generalization of estimates from site $j_{1}$ to $j_{2}$ for all pairs $j_{1},j_{2}\in \left\{1,\cdots ,N\right\}$, $j_{1}\neq j_{2}$, in each application. In general, we call the population in site $j_{1}$ as the source population P and the population in site $j_{2}$ as the target population Q. As typically the case in practice, for a generalization task, we assume all data from P are observed while only covariates X are observed from Q. When we evaluate the performance of various generalization estimators, we will use the full data in the target population Q to empirically evaluate how well the generalization estimators approximate the benchmark estimates in Q.

### 2.3. Challenge: Covariate Shift Cannot Explain Away Distributional Shift.

The vast majority of existing methods for generalization assume that accounting for distributional shifts in observed covariates is sufficient, known as the covariate shift assumption. For example, when researchers want to generalize causal effects in one site to another site in the Pipeline project, they may assume that adjusting for observed characteristics of respondents, such as political ideology, gender, age, and education, is sufficient for generalization (consistent estimation and valid inference for the parameter in the target site).

**Fig. 3.**
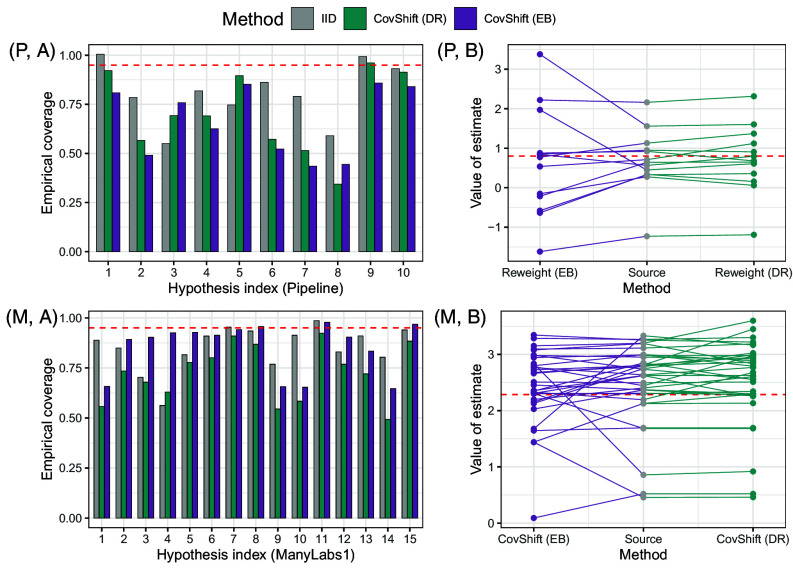
Insufficient explanatory role of covariate shift. *Left*: Undercoverage of 95% prediction intervals based on the i.i.d. assumption (gray) and covariate shift assumption adjusted via doubly robust estimator (green) and entropy balancing (purple), averaged over all pairs of sites within each hypothesis for the Pipeline project (*P*, *A*) and the ManyLabs 1 data (*M*, *A*), respectively. The red dashed line is the nominal level. *Right*: Estimates based on existing approaches (via doubly robust estimator (green) and entropy balancing (purple)) do not bring the source estimates (gray) closer to the target estimate (red dashed line). As illustrative examples, we show results when generalizing from all other sites to site 5 (raw ID) in hypothesis 5 in the Pipeline data (*P*, *B*) and when generalizing from all other sites to site 4 in hypothesis 4 in ManyLabs 1 data (*M*, *B*). The segments connect estimates for the same pairs of sites.

However, in line with recent empirical evaluations (13–15), we find that this common assumption of covariate shift is often insufficient to explain away distributional shifts in the real-world applications. Fig. 3 examines existing procedures that adjust for shift in observed covariates. We consider generalizing treatment effects from one site to another, using two commonly used estimators—the doubly robust (DR) estimator (11, 26) and the entropy balancing (EB) estimator (28, 50)—to construct point estimates that are consistent for the target parameter under the covariate shift assumption. Then, we follow ref. 51 to construct prediction intervals that would cover the target estimator with probability 1−α under covariate shift, and evaluate their empirical coverage.^†^ As a simple baseline, we also compute prediction intervals based on the i.i.d. assumption that assumes no distribution shift between sites. Detailed estimation procedures are deferred to *SI Appendix*, section 2.B. and we include a brief overview here. When generalizing from site $j_{1}$ to $j_{2}$ for hypothesis k, the two effect sizes are ${\hat{\theta }}_{j_{1}}^{\left(k\right)}$ and ${\hat{\theta }}_{j_{2}}^{\left(k\right)}$, respectively. The prediction interval by the IID method centers around ${\hat{\theta }}_{j_{1}}^{\left(k\right)}$ based on the asymptotics ${\hat{\theta }}_{j_{2}}^{\left(k\right)}-{\hat{\theta }}_{j_{1}}^{\left(k\right)}∼N\left(0,\sigma_{\mathrm{iid}}^{2}\right)$, where $\sigma_{\mathrm{iid}}^{2}$ can be consistently estimated if $P_{j_{1}}^{\left(k\right)}=P_{j_{2}}^{\left(k\right)}$. The CovShift methods compute an estimator ${\hat{\theta }}_{j_{1}\to j_{2}}^{\left(k\right)}$ using reweighting, and the prediction interval centers around ${\hat{\theta }}_{j_{1}\to j_{2}}^{\left(k\right)}$ based on the asymptotic distribution ${\hat{\theta }}_{j_{2}}^{\left(k\right)}-{\hat{\theta }}_{j_{1}\to j_{2}}^{\left(k\right)}∼N\left(0,\sigma_{\mathrm{cs}}^{2}\right)$ for some constant $\sigma_{\mathrm{cs}}^{2}$ that can be consistently estimated under the covariate shift assumption. We then evaluate the coverage of these prediction intervals for ${\hat{\theta }}_{j_{2}}^{\left(k\right)}$ among all pairs $\left(j_{1},j_{2}\right)$.

Fig. 3 highlights two key findings:

(i) Adjusting for distribution shift is necessary, as prediction intervals based on the assumption of no distribution shift (denoted as IID) do not deliver valid coverage (gray bars).
(ii) The explanatory role of covariate shift is insufficient. This is evident from the undercoverage in panel (*A*) of both of the two CovShift methods. The coverage is sometimes even lower than IID; this is because the uncertainty that remains after adjustment is underestimated. When comparing the estimates in the source population and generalization estimates in panel (*B*), we see that adjusting for covariate shift does not necessarily bring the estimators closer to the target estimate.

## 3. The Predictive Role of Covariate Shift

In this paper, we highlight a role of covariate shifts: Observed covariate shifts can be used to predict unobserved shifts in the conditional distribution of Y given X, even though covariate shifts cannot fully explain the total distributional shift. We first propose standardized measures of distributional shifts, and then provide empirical and theoretical evidence for the predictive role of covariate shift.

### 3.1. Comparing the Strength of Covariate Shift and Conditional Shift.

We begin by defining our measures of the two sources of distribution shifts: i) the covariate shift in X (the part commonly addressed in existing methods) and ii) the conditional shift—the shift in the conditional distribution of Y given X (the part assumed away under the covariate shift assumption). Our approach is based on two simple principles:

- Scale invariance. We would like our measures to reflect the strength of perturbations to the probability space, hence they should be invariant to scalings of the variables.
- Numerical stability. We would like our measures to be useful in guiding real generalization tasks, hence they should permit stable estimation.

Throughout the paper, we suppose the goal is to understand how causal effects change across sites, and we have two randomized experiments with treatment assignment probability π (most studies in our datasets are of this form). We can write the difference in the causal effects as

$$\begin{array}{cc} \theta_{Q}-\theta_{P} & =E_{Q}\left[\phi \left(T,Y\right)\right]-E_{P}\left[\phi \left(T,Y\right)\right]\\ \text{where}\;\phi & =\frac{T}{\pi }Y-\frac{1-T}{1-\pi }Y, \end{array}$$

where $E_{P}$ and $E_{Q}$ are expectations over the source and target distribution. While we focus our discussion on causal effects in this paper for the sake of clear presentation, our proposed approach is applicable to any parameter of interest by redefining ϕ. For example, some studies in the Pipeline project use a one-sample t test, in which case the parameter of interest is the mean of the outcome and ϕ=Y.

We begin by conceptually decomposing the impact of overall distribution shift on the parameter of interest ($\theta_{Q}-\theta_{P}$) to measure the shifts in X and ϕ given X separately:

[1] $$\begin{array}{cc} & E_{Q}\left[\phi \right]-E_{P}\left[\phi \right]\\ & =\underset{=:\;\text{Covariate shift}}{\underbrace{\{E_{Q}\left[\phi_{P}\left(X\right)\right]-E_{P}\left[\phi_{P}\left(X\right)\right]\}}}+\;\underset{=:\;\text{Conditional shift}}{\underbrace{\{E_{Q}\left[\phi_{Q}\left(X\right)-\phi_{P}\left(X\right)\right]\}}}, \end{array}$$

where $\phi_{P}\left(X\right):=E_{P}\left[\phi |X\right]$ (resp. $\phi_{Q}\left(X\right):=E_{Q}\left[\phi |\;X\right]$) is the conditional expectation of ϕ in the source (resp. target) distribution. When the parameter of interest is the ATE, we have $\phi_{P}\left(X\right)=E_{P}\left[Y\left(1\right)-Y\left(0\right)|X\right]$, the conditional ATE. In ref. 14, the decomposition Eq. **1** is used to diagnose the roles of different distribution shifts on the discrepancy of effect estimates between a pair of studies.

The first “Covariate shift” term in the decomposition Eq. **1** captures the shift in the observed covariates X. Intuitively, it measures how much the estimate can be brought closer to the target by adjusting for the shift in X. This term becomes larger when the strength of shift between P(X) and Q(X) is larger. Importantly, it also depends on the heterogeneity in $\phi_{P}\left(X\right)$, that is, how much the parameter of interest varies with the covariates. Our proposed distribution shift measures will remove the impact of such heterogeneity (sensitivity) on our measure of the strength of distribution shift to ensure interpretability and scale invariance.

The second term in Eq. **1**, $E_{Q}\left[\phi_{Q}\left(X\right)-\phi_{P}\left(X\right)\right]$, captures the shift in the conditional expectation E[ϕ|X] between the source and target distribution. For example, when the parameter of interest is the ATE, this part captures how much the conditional ATE changes between the source and target distribution. Similarly, it not only depends on the strength of conditional shift but also the heterogeneity in $\phi -\phi_{P}\left(X\right)$; again, the latter will be removed in our measures.

The common assumption of covariate shift essentially assumes away the second shift in the conditional distribution. We formalize it as follows.

Assumption 1 *[Covariate Shift]* P(ϕ|X)=Q(ϕ|X) *holds* $P_{X}$-*almost surely*.

If ϕ=Y, this assumption is the classical covariate shift assumption in machine learning. For experiments, *Assumption* 1 is satisfied if the treatment probabilities do not change and the conditional distribution of the potential outcomes is invariant, i.e., if P(Y(1),Y(0)|X)=Q(Y(1),Y(0)|X).

*Assumption* 1 implies the second term in Eq. **1** is zero, and thus it suffices to adjust for the shift in observed covariates (the first term). While this is a commonly imposed assumption for the identifiability of target parameters, as discussed in Section 2.3, it is often violated in practice, which implies that the conditional shift (the second term) is often nonzero in real-world applications. Therefore, instead of assuming away the conditional shift, we are to carefully investigate the relationship between the two shifts to offer insights for moving beyond the covariate shift assumption.

We define distribution shift measures by rescaling the two terms in Eq. **1** by their SD for scale invariance:

[2] $$\begin{array}{cc} & \text{Relative conditional shift}=\frac{E_{Q}\left[\phi -\phi_{P}\left(X\right)\right]}{{\text{sd}}_{P}\left(\phi -\phi_{P}\left(X\right)\right)}, \end{array}$$

[3] $$\begin{array}{cc} & \text{Relative covariate shift}=\frac{|E_{Q}\left[\phi_{P}\left(X\right)\right]-E_{P}\left[\phi_{P}\left(X\right)\right]|}{{\text{sd}}_{P}\left(\phi_{P}\left(X\right)\right)}. \end{array}$$

We will measure the strength of the conditional shift by the “relative conditional shift” Eq. **2**. However, an issue with the “relative covariate shift” measure Eq. **3** is numerical instability whenever ${\text{sd}}_{P}\left(\phi_{P}\left(X\right)\right)$ is close to zero. This might be problematic in social science applications where the explanatory power of covariates X can be low. To address this issue, we will use a Mahalanobis-type, “stabilized” measure:

[4] $$\begin{array}{cc} & \text{Stabilized covariate shift measure}\\ & =\sqrt{\frac{1}{L}\sum_{\ell =1}^{L}\frac{{\left(E_{Q}\left[X_{\ell }\right]-E_{P}\left[X_{\ell }\right]\right)}^{2}}{{\text{Var}}_{P}\left(X_{\ell }\right)}}, \end{array}$$

where L is the number of covariates. Here, we assume the X is whitened (based on P) before computing, so the $X_{\ell }$’s are uncorrelated. With correlated features, Eq. **4** would have to be replaced by the Mahalanobis distance. We justify this covariate shift measure from a theoretical perspective in Section 3.3. Importantly, this measure is also invariant under the scaling of features.

Remark 1 We note that both i) rescaling by SD for scale invariance and ii) adopting stabilized measure of covariate shift are crucial for interpretable and robust empirical insights. We illustrate the importance of these considerations through an ablation study in *SI Appendix*, section 4 which explores alternative distribution shift measures without these elements. These alternative distribution shift measures either fail to induce the predictive role or lead to much wider intervals in generalization due to numerical instability.

In our evaluations, the two population measures will be replaced by their estimators (52). The estimation details are deferred to *SI Appendix*, section 2 with brief summary and specific references in the corresponding parts of the paper.

### 3.2. Empirical Evidence: Covariate Shift Can Bound Conditional Shift.

Using data from both the Pipeline project and the ManyLabs1 project, we establish empirical evidence that with our distribution shift measures, the covariate shift can *bound* the conditional shift, even though the strength of both may change across hypotheses and sites. Because the covariate shift is estimable in common generalization tasks, researchers can use this bounding relationship to predict the conditional shift, which is usually unobserved. We provide theoretical justification for the empirical findings in the next subsection.

We estimate the two distribution shift measures for any pair of sites for each hypothesis in the Pipeline project and the ManyLabs1 project. For any given hypothesis k, we define ϕ following the original analysis (c.f. *SI Appendix*, Tables S2 and S4 for details), and $P=S_{j_{1}}^{\left(k\right)}$, $Q=S_{j_{2}}^{\left(k\right)}$ for all site pairs $\left(j_{1},j_{2}\right)$ and hypothesis index k. Then, we compute an estimate for the relative conditional shift (denoted by ${\hat{t}}_{Y|X}^{j_{1}\to j_{2},\left(k\right)}$), and an estimate for the relative covariate shift (denoted by ${\hat{t}}_{X}^{j_{1}\to j_{2},\left(k\right)}$), where $E_{Q}\left[\phi_{P}\left(X\right)\right]$ is estimated by the same point estimate ${\hat{\theta }}_{j_{1}\to j_{2}}^{\left(k\right)}$ used in Section 2.3, and we develop doubly robust estimators for the denominators ${\text{sd}}_{P}\left(\phi -\phi_{P}\left(X\right)\right)$ and ${\text{sd}}_{P}\left(\phi_{P}\left(X\right)\right)$. Other quantities in Eqs. **2** and **3** are estimated by sample average. The estimation details are in *SI Appendix*, section 2.C.

**Fig. 4.**
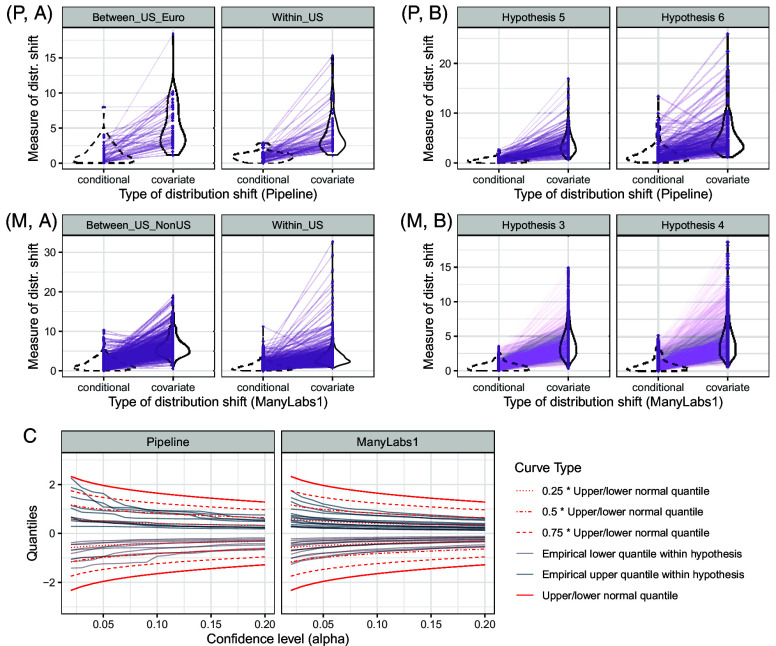
Our covariate shift measures bound conditional shift measures in various contexts (pivotality). *Left*: Conditional and covariate shift measures for site pairs between United States and Europe/Non–United States and site pairs within United States in the Pipeline data (*P*, *A*) and the ManyLabs 1 data (*M*, *A*). *Right*: Conditional and covariate shift measures for all site pairs in hypotheses 5 and 6 in Pipeline (*P*, *B*), and those in hypotheses 3 and 4 in ManyLabs 1 (*M*, *B*). A few (≤5) largest values are removed for visualization. Panel (*C*): Empirical quantiles of the ratios between conditional and covariate shift measures within each hypothesis (grey and brown curves). The red curves are multiples of the quantiles of standard Gaussian for reference.

Fig. 4 compares the conditional shift measure ${\hat{t}}_{Y|X}^{j_{1}\to j_{2},\left(k\right)}$ and the covariate shift measure ${\hat{t}}_{X}^{j_{1}\to j_{2},\left(k\right)}$ in various contexts. The *Left* two panels (*P*, *A*) and (*M*, *A*) show site pairs $\left(j_{1},j_{2}\right)$, where one is in the United States and the other is not in the United States, as well as pairs where both sites are in the United States. The *Right* two panels (*P*, *B*) and (*M*, *B*) show site pairs within two hypotheses for each project.

In (*P*, *A*) and (*M*, *A*), the distribution shift between US–Non-US pairs tends to be larger than within–United States pairs. In (*P*, *B*) and (*M*, *B*), the magnitude of distribution shifts also vary across hypotheses. Despite the variation across contexts, however, the covariate shift measure upper bounds the conditional shift measure most of the time. In addition, when the conditional shift is larger (which is typically unobservable in a generalization task), the observable covariate shift also tends to be larger, justifying the “predictive role” of the covariate shift for the conditional shift.

Finally, panel (*C*) of Fig. 4 provides a more quantitative illustration of the predictive role. In the figure, each curve is the α/2 or (1−α/2)-th empirical quantiles of the ratios ${\left\{{\hat{t}}_{Y|X}^{j_{1}\to j_{2},\left(k\right)}/{\hat{t}}_{X}^{j_{1}\to j_{2},\left(k\right)}\right\}}_{j_{1}\neq j_{2}}$ for a hypothesis k across a series of confidence levels α on the x-axis. For reference, we compare them with multiples of standard normal distribution quantiles. A few comments are in order:

- First, the bounding relationship $|{\hat{t}}_{Y|X}^{j_{1}\to j_{2},\left(k\right)}|\leq {\hat{t}}_{X}^{j_{1}\to j_{2},\left(k\right)}$ holds with high probability. Thus, in practice, the belief that $|{\hat{t}}_{Y|X}|\leq {\hat{t}}_{X}$ is a plausible option to establish a range of the conditional shift strength. We will see reliable effect generalization based on this idea in Section 4.
- Second, if one wants to adjust the upper bound of $|{\hat{t}}_{Y|X}^{j_{1}\to j_{2},\left(k\right)}|/{\hat{t}}_{X}^{j_{1}\to j_{2},\left(k\right)}$ based on a desired confidence level, it is reasonable to use some multiplicative of standard normal, e.g., 0.75. Indeed, the empirical quantiles are smooth and similar to normal quantiles in general. This suggests a “smooth” and “random” nature of distribution shift, instead of being adversarial.

### 3.3. Theoretical Analysis: Random Distribution Shift Model.

We offer a theoretical framework to motivate the predictive role of covariate shift, justifying the empirical evidence in the last section.

We begin by modeling the data collection procedure as a two-stage process. In the first stage, the underlying distribution is randomly perturbed. This perturbation aims to model unintended changes in the population or deviations from the experimental protocols despite efforts to keep them, etc. In the second, data are drawn i.i.d. from the perturbed distributions. This leads to three sources of uncertainty:

$$\begin{array}{cc} & \underset{\text{estimator on target dataset}}{\underbrace{{\hat{\theta }}_{Q}}}-\underset{\text{estimator on source dataset}}{\underbrace{{\hat{\theta }}_{P}}}\\ & =\underset{\text{sampling uncertainty}}{\underbrace{{\hat{\theta }}_{Q}-\theta \left(Q\right)}}+\underset{\text{random shift}}{\underbrace{\theta (Q)-\theta (P)}}+\underset{\text{sampling uncertainty}}{\underbrace{\theta \left(P\right)-{\hat{\theta }}_{P}}}. \end{array}$$

Here, “sampling uncertainty” refers to the usual statistical uncertainty arising from randomly drawing observations from an underlying population P or Q, and “random shift” refers to the discrepancy between two underlying distributions P and Q due to natural “perturbations”. In the following, we construct Q by randomly perturbing P. Constructing P by randomly perturbing Q or constructing P and Q by randomly perturbing a third $P^{0}$ would lead to the same asymptotics.

Our model for distribution shift includes three elements:

- We assume that the treatment distribution is invariant, since the treatment probability is fixed and chosen by the scientists for the datasets we consider here.
- There is distribution shift in observed covariates X, which we model as random. There is shift in some unobserved effect modifiers U, modeled as random, too.
- The outcome Y is a function of treatment indicator T, covariates X, and unobserved modifiers U. Thus, the shift in U is the driving factor for the conditional shift.

Let Y=g(T,X,U), where the treatment T is independent of the modifiers (X,U) under P due to randomization. Recall that X is observed, while U is not.

#### 3.3.1. Random distribution shift.

The key idea of our random distribution shift model is that the original probability measure is randomly brought up and down in small pieces which, put together, lead to CLT-like behavior of the estimates with inflated variance. To be precise, we let events ${\left\{C_{m}^{\left(M\right)}\right\}}_{m=1}^{M}$ be a disjoint covering of the sample space of (X,U). We assume that these “pieces” have the same probability mass, i.e., $P(C_{m}^{\left(M\right)})=1/M$ for m=1,…,M. Later, we will take M→∞ to describe a scenario where many random factors change the probability masses of $C_{m}^{\left(M\right)}$ independently.

Our model describes random perturbations of P in these small event pieces. Specifically, we define the randomly reweighted distribution Q for any event $E\subseteq \cup_{m=1}^{M}C_{m}^{\left(M\right)}$ via

[5] $$\begin{array}{c} Q\left(E\right)=\sum_{m=1}^{M}P\left(E|\;C_{m}^{\left(M\right)}\right)\cdot \frac{W_{m}}{\frac{1}{M}\sum_{m^{\prime }=1}^{M}W_{m^{\prime }}}, \end{array}$$

where ${\left(W_{m}\right)}_{m=1}^{M}$ are i.i.d. positive random variables that are bounded away from zero and have finite variance. As written above, the treatment indicator T is assumed to be independent of the modifiers (X,U) under both P and Q, and its distribution is invariant.

**Fig. 5.**
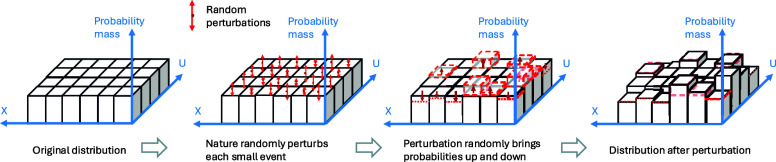
Visualization of the random distribution shift model. The original distribution is randomly perturbed to produce the distribution from which data are i.i.d. drawn. Our model assumes independent perturbation/reweighting of equal-probability small events and takes the number of small events to infinity.

Fig. 5 visualizes this idea, where probability masses of small events $\left\{C_{m}\right\}$ are independently perturbed by “nature.” Such small, random perturbations are suitable to describe unintended but inevitable distribution shifts in such multisite replication studies, such as unintended changes in the study population or random deviations from the experimental protocols despite efforts to keep them, etc. We make the random distribution shift model explicit as follows.

Assumption 2 *The outcome is an unknown function* Y=g(X,T,U) *of the treatment* T∈{0,1}, *observed covariates* X, *and unobserved modifiers* U.

Assumption 3 *Let events* ${\left\{C_{m}^{\left(M\right)}\right\}}_{m=1}^{M}$ *be a disjoint covering of the sample space of* (X,U), *and* $P(C_{m}^{\left(M\right)})=1/M$ *for* m=1,⋯,M. *We assume that the step functions on these pieces approximate square-integrable functions*.^‡^ *The target distribution* Q *is obtained by randomly perturbing* P *according to Eq. **5**. In addition, the distribution of* T *is independent of* (X,U) *under both* P *and* Q, *and* P(T=1)=Q(T=1).

Making the grid fine-grained and taking limits ($n_{Q},n_{P},M\to \infty$) we obtain a distributional CLT that describes the shift of empirical means under this two-stage sampling procedure. There are various asymptotic regimes that one could consider. Considering the asymptotic regime where $n_{Q}/M\to \rho \in \left(0,\infty \right)$ means sampling uncertainty and distributional uncertainty are of the same order (19). Taking $n_{Q}/M\to 0$ means distributional uncertainty is of larger order than sampling uncertainty (20). In the following, we focus on scenarios where sampling uncertainty and distributional uncertainty are of the same order as we let M→∞.

Assumption 4 *The sample sizes obey* $n_{Q}/M\to r_{Q}$ *and* $n_{P}/M\to r_{P}$ *for some constants* $r_{P},r_{Q}>0$ *as* M→∞.

Theorem 1 (Distributional CLT). *Let* ${\hat{E}}_{Q}\left[\psi \right]$ *denote the sample mean of a function* ψ(T,X,U) *over* $n_{Q}$ *i.i.d. draws from* Q *and* ${\hat{E}}_{P}\left[\psi \right]$ *denote the sample mean of* ψ *over* $n_{P}$ *i.i.d. draws from* P. *Under* *Assumptions 2*, *3*, *and* *4*, *for any function* $\psi \left(T,X,U\right)\in L^{2}\left(P\right)$, *we have*

$$\begin{array}{c} s_{n}^{-1}\left({\hat{E}}_{Q}\left[\psi \right]-{\hat{E}}_{P}\left[\psi \right]\right)\overset{d}{\to }N\left(0,1\right), \end{array}$$

*where* $s_{n}^{2}=\left(\frac{1}{n_{P}}+\frac{1}{n_{Q}}\right){\text{Var}}_{P}\left(\psi \right)+\delta_{\text{M}}^{2}{\text{Var}}_{P}\left(E_{P}\left[\psi |X,U\right]\right)$, *and* $\delta_{\text{M}}^{2}=\frac{1}{M}\frac{E[W^{2}]}{E{\left[W\right]}^{2}}$ *measures the strength of perturbation. If* ψ *is a vector of functions, then* ${\text{Var}}_{P}\left(\psi \right)$ *and* ${\text{Var}}_{P}\left(E_{P}\left[\psi |X,U\right]\right)$ *are covariance matrices*.

In *Theorem* 1, the variance term $\left(\frac{1}{n_{P}}+\frac{1}{n_{Q}}\right){\text{Var}}_{P}\left(\psi \right)$ is the usual asymptotic variance one would obtain under the i.i.d. assumption that P=Q. In addition, random perturbations to the distributions contributes a factor of $\delta_{\text{M}}^{2}{\text{Var}}_{P}\left(E_{P}\left[\psi |X,U\right]\right)$, where only the variance of $E_{P}\left[\psi |X,U\right]$ counts because only the distribution of (X,U) is perturbed, while that of T remains invariant.

*Theorem* 1 describes a marginal perspective that includes two sources of randomness: i) the sampling uncertainty, i.e., the observations being i.i.d. samples from P and Q, respectively, and ii) the distributional uncertainty, where Q is randomly perturbed from P described by the random distribution shift model. Conditional on the randomness of distribution shift, the empirical averages ${\hat{E}}_{P}\left[\psi \right]$ and ${\hat{E}}_{Q}\left[\psi \right]$ center around distinct values θ(P) and θ(Q), respectively. Similar perspectives can be found in random effect models commonly used in meta-analysis (53, 54) and analysis of heterogeneity in replication studies (40), where each study is assumed to be drawn from a population of studies. Compared with these models, our model is nonparametric, and the symmetric structure allows estimation and generalization with only one dataset (e.g., leveraging the predictive role).

Remark 2 We assume that the treatment distribution is invariant in well-controlled experiments. For conceptual clarity, treatment compliance is implicitly assumed to be perfect. While extensions of the random shift model to instrumental variable settings might be feasible (e.g., the compliance pattern shifts across studies), this requires careful empirical justification and theoretical development.

#### 3.3.3. Why covariate shift often upper bounds conditional shift.

We now further discuss how this distributional CLT implies that covariate shift often upper bounds conditional shift.

For simplicity, we focus on deriving the generalization error for the estimators ${\hat{\theta }}_{P}={\hat{E}}_{P}\left[\phi \right]$ and ${\hat{\theta }}_{Q}={\hat{E}}_{Q}\left[\phi \right]$. A formal justification of this influence function approximation for general M-estimators can be found in ref. 19. The numerator of our relative conditional shift measure Eq. **2** equals the difference-in-means estimator with $\psi =\phi -\phi_{P}\left(X\right)$ (ignoring the estimation of $\phi_{P}\left(X\right)$ for simplicity), where $\phi =\frac{T}{\pi }Y-\frac{1-T}{1-\pi }Y$ or ϕ=Y depending on the hypothesis. With the distributional CLT, the squared relative conditional shift measure obeys

[6] $$\begin{array}{c} {\left[\hat{2}\right]}^{2}\overset{d}{=}(\frac{1}{n_{P}}+\frac{1}{n_{Q}}+\delta_{M}^{2}\frac{{\text{Var}}_{P}\left(E_{P}\left[\psi |X,U\right]\right)}{{\text{Var}}_{P}\left(\psi \right)})Z_{1}+o_{P}\left(\delta_{M}\right), \end{array}$$

where $Z_{1}∼\chi^{2}\left(1\right)$. Using the distributional CLT for the covariates (taking $\psi =X_{\ell }$), we obtain that standardized squared differences follows a scaled chi-square distribution:

[7] $$\begin{array}{c} \frac{{\left({\hat{E}}_{Q}\left[X_{\ell }\right]-{\hat{E}}_{P}\left[X_{\ell }\right]\right)}^{2}}{{\hat{\text{Var}}}_{P}\left(X_{\ell }\right)}\overset{d}{=}(\frac{1}{n_{P}}+\frac{1}{n_{Q}}+\delta_{M}^{2})Z_{1}+o_{P}\left(\delta_{M}\right). \end{array}$$

Here, ${\hat{\text{Var}}}_{P}\left(X_{\ell }\right)$ is the sample variance of $X_{\ell }$ in P. Thus, up to lower-order terms, Eq. **6** is stochastically smaller than Eq. **7** because ${\text{Var}}_{P}\left(E_{P}\left[\psi |X,U\right]\right)/{\text{Var}}_{P}\left(\psi \right)\leq 1$. In other words, the standardized conditional shift is stochastically smaller than the standardized covariate shift. This is in line with the empirical phenomenon in Fig. 4. It also justifies replacing Eq. **3** by the stabilized Eq. **4**, roughly because the perturbations are homogeneous in different directions. If we average over multiple uncorrelated covariates $X_{\ell }$, by the distributional CLT, the squared covariate shift measure obeys

[8] $$\begin{array}{c} {\left[\hat{4}\right]}^{2}\overset{d}{=}\left(\frac{1}{n_{P}}+\frac{1}{n_{Q}}+\delta_{\text{M}}^{2}\right)\frac{Z_{L}}{L}+o_{P}\left(\delta_{M}\right), \end{array}$$

where $Z_{L}∼\chi^{2}\left(L\right)$. As $\frac{Z_{L}}{L}\to 1$ for L→∞, Eq. **8** will be close to $\frac{1}{n_{P}}+\frac{1}{n_{Q}}+\delta_{\text{M}}^{2}$. In our empirical studies, the covariates exhibit low correlation, hence we directly employ the formula Eq. **4**.

We remark that our model is one of the potential models that may explain the empirical finding. In *SI Appendix*, section 5.A, we further contextualize the insights under our model by connecting it to random-effect-type models commonly used in meta-analysis (53, 54) and heterogeneity analysis (40). There, we show that a similar pattern may arise if random parameters specifying the distributions of observed covariates and hidden effect modifiers are of comparable variances, and discuss the benefits of using the current nonparametric model.

These results motivate using a ratio of the estimated conditional shift and estimated covariate shift as a pivot to create prediction intervals. In the next, we propose such prediction intervals and evaluate the empirical performance.

## 4. Effect Generalization Exploiting the Predictive Role

In this section, we demonstrate that leveraging the predictive role of covariate shift leads to reliable generalization for target distributions. To this end, we build prediction intervals^§^ for the target population estimate ${\hat{\theta }}_{Q}$ based on our distribution shift measure and evaluate their empirical coverage.

### 4.1. Constructing Prediction Intervals.

Before presenting the results, we begin with a high-level overview of our prediction intervals based on the predictive role, while we defer technical details on the estimation procedures to *SI Appendix*, section 2.D.

We consider generalization tasks where a scientist has access to full observations from the source distribution P but only the covariates X from the target distribution Q. To construct our prediction interval for the target *estimate* ${\hat{\theta }}_{Q}$, we leverage the ratio between the covariate and conditional shift measures:

$$\begin{array}{c} \hat{r}:={\hat{t}}_{Y|X}/\;{\hat{t}}_{X}, \end{array}$$

where ${\hat{t}}_{Y|X}$ is the estimated conditional shift measure Eq. **2**, and ${\hat{t}}_{X}$ is the covariate shift measure Eq. **4**. Note that one can estimate ${\hat{t}}_{X}$ but not ${\hat{t}}_{Y|X}$ in a generalization task. Suppose the distribution of $\hat{r}$ can be characterized (e.g., using approaches we discuss below) so that one can find some $\left(r_{L},r_{U}\right)$ obeying approximately

$$\begin{array}{c} P(r_{L}\leq \hat{r}\leq r_{U})\geq 1-\alpha . \end{array}$$

By definition, inverting the above event leads to a general form of our prediction interval for ${\hat{\theta }}_{Q}$:

[9] $$\begin{array}{c} \hat{C}=\left[{\hat{\theta }}_{w}+r_{L}\cdot {\hat{t}}_{X}\cdot {\hat{s}}_{Y|X},\;{\hat{\theta }}_{w}+r_{U}\cdot {\hat{t}}_{X}\cdot {\hat{s}}_{Y|X}\right], \end{array}$$

where ${\hat{s}}_{Y|X}$ is an estimate for ${\text{sd}}_{P}\left(\phi -\phi_{P}\right)$ in Eq. **2**, and ${\hat{\theta }}_{w}$ is an estimator for $E_{Q}\left[\phi_{P}\left(X\right)\right]$ in Eq. **2** which adjusts for the covariate shift. Above, all quantities in Eq. **9** except $r_{L}$ and $r_{U}$ can be estimated with full observations from the source distribution P and the covariate data from the target distribution Q. In each part of the empirical evaluation in Section 4.2, we will further expand on how $\left(r_{L},r_{U}\right)$ is calibrated and the specific choice of sites (P,Q) to be evaluated.

**Fig. 6.**
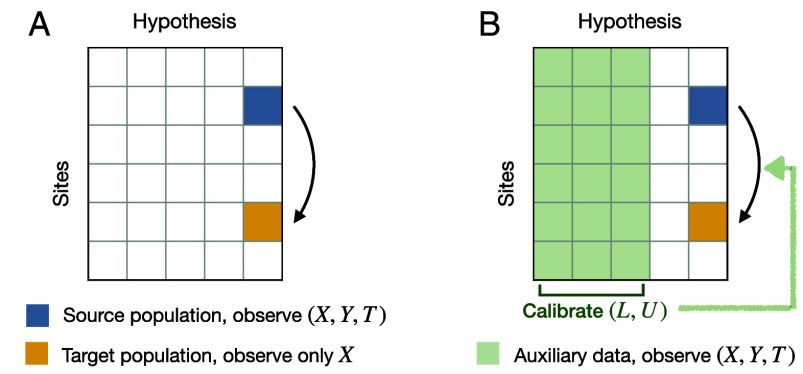
Generalization in two scenarios for the availability of data. Panel (*A*): Generalization without auxiliary data from source (blue) to target (yellow). Panel (*B*): Generalization with auxiliary data (green) from the same sites for other hypotheses. The auxiliary data are used to calibrate L and U for a new generalization task.

We consider two ways to calibrate $\left(r_{L},r_{U}\right)$ under two scenarios of data availability (visualized in Fig. 6):

1. Constant calibration. We construct prediction intervals assuming the conditional shift measure is bounded by the covariate shift measure [constant bounds $\left(r_{L},r_{U}\right)=\left(-1,1\right)$]. This is theoretically justified under the random distribution shift model (Section 3.3). This approach is applicable to a generalization task with no information other than covariate data from the target site.
2. Data-adaptive calibration. We construct prediction intervals by calibrating the relative strengths of conditional and covariate shift measures using existing data. This is applicable when some relevant auxiliary data are available (but not full observations in the target site) and we believe they inform the (relative) strengths of distribution shifts in the current generalization task.

Of course, the set of available data in the second approach can be flexible; we explore other scenarios in *SI Appendix*, section 3.A. These prediction intervals are compared with three baselines:

1. IID. Prediction intervals under the i.i.d. assumption, i.e., P=Q, ignoring distribution shift (same as Section 2.3).
2. WorstCase. Prediction intervals based on worst-case bounds under restrictions on the distributional distance between the target distribution and the reweighted distribution, i.e., $\text{KL}(Q_{X}⊗P_{Y|X}\|Q)\leq \rho$, where ρ is calibrated with data (see *SI Appendix*, section 1.D for details).
3. Oracle. Prediction intervals calibrated with true knowledge of the relative strength of covariate shift and conditional shift measures. This is the “ideal” but unrealistic version of our method.

We evaluate the generalization performance of different methods by the empirical coverage and average length of prediction intervals across all site pairs for each hypothesis.

### 4.2. Empirical Evaluation.

#### 4.2.1. Without any auxiliary data.

In the first scenario, the scientists have data from the source distribution, but they do not have any information other than covariates X from the target distribution. In this setting, researchers can use our proposed approach with constant calibration.

More specifically, we consider the generalization of site $j_{1}$ to $j_{2}$ for all pairs $j_{1},j_{2}\in \left\{1,\cdots ,N\right\}$, $j_{1}\neq j_{2}$, for each hypothesis k∈{1,…,K} in each application. When we construct prediction intervals, we assume all data from $j_{1}$ are observed while only covariates X are observed from $j_{2}$. When we then *evaluate* the statistical performance of various generalization methods, we use the full data in site $j_{2}$ to empirically evaluate how well each method covers the benchmark estimate in $j_{2}$. The Oracle method takes $r_{L},r_{U}$ in Eq. **9** as the empirical (α/2,1−α/2)-th quantile of ${\left\{{\hat{t}}_{Y|\;X}^{j_{1}\to j_{2},\left(k\right)}\right\}}_{j_{1}\neq j_{2}}$ for α=0.05. Ours_Const sets $\left(r_{L},r_{U}\right)=\left(-1,1\right)$. WorstCase sets ρ as the 0.99-th quantile (replacing maximum for stability) of the estimated KL divergence among all pairs $j_{1}\neq j_{2}$, and compute lower/upper bounds for the *parameter* θ(Q) with distributionally robust optimization (55), i.e., under the constraint $\text{KL}(Q_{X}⊗P_{Y|X}\|Q)\leq \rho$.^¶^

**Fig. 7.**
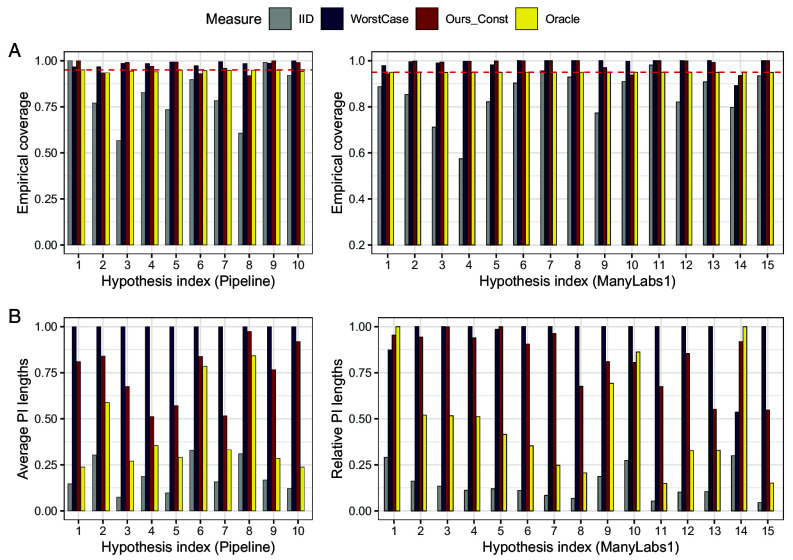
Effect generalization without auxiliary data. Row (*A*): Empirical coverage of prediction intervals via constant calibration at nominal level 1−α=0.95 and three baseline methods using the Pipeline data (*Left*) and ManyLabs 1 data (*Right*). Row (*B*): Average length of prediction intervals for constant calibrated prediction intervals at nominal level 1−α=0.95 for the four methods, normalized by the largest average length, using the Pipeline (*Left*) and ManyLabs 1 (*Right*) data.

In Fig. 7, we report the empirical coverage and relative lengths of prediction intervals averaged over all pairs within each hypothesis. Across two distinct applications, our procedure (denoted as Ours_Const) achieves valid 95% coverage in most cases (*A*). WorstCase prediction intervals achieve the target coverage as well but are much wider than the proposed intervals (*B*). Not surprisingly, intervals based on the i.i.d. assumption exhibit undercoverage.

#### 4.2.2. With auxiliary data.

Next, we examine how we can improve the performance of our estimator when researchers use some auxiliary data to adaptively calibrate our method. We consider a scenario where data from all sites exist for other hypotheses to build prediction intervals for a new hypothesis. In practice, this arises when there are existing data from the same set of sites on other research questions or hypotheses.

We randomly sample $K_{\mathrm{exist}}\subseteq \left\{1,\cdots ,K\right\}$ as “existing hypotheses” with full observations, and $K_{\mathrm{eval}}=\left\{1,\cdots ,K\right\}\backslash K_{\mathrm{exist}}$ as “future hypotheses” whose generalization is to be evaluated. For each $k\in K_{\mathrm{eval}}$, Oracle takes the (α/2,1−α/2)-th empirical quantile of ${\left\{{\hat{t}}_{Y|\;X}^{j_{1}\to j_{2},\left(k\right)}\right\}}_{j_{1}\neq j_{2}}$ for α=0.05. Ours sets $\left(r_{L},r_{U}\right)$ as the (α/2,1−α/2)-th empirical quantile of ${\left\{{\hat{t}}_{Y|\;X}^{j_{1}\to j_{2},\left(k\right)}\right\}}_{j_{1}\neq j_{2}}^{k\in K_{\mathrm{exist}}}$. WorstCase is similar to that in Section 4.2.1, where ρ is the 0.99-th empirical quantile of the estimated KL-divergences among all pairs $j_{1}\neq j_{2}$ and $k\in K_{\mathrm{exist}}$. We then evaluate the empirical coverage of these prediction intervals for $k\in K_{\mathrm{eval}}$. To ensure stable evaluation, the ordering of the sites is randomly permuted for 10 times. Additional calibration scenarios (generalizing to new sites for existing hypotheses and new sites for new hypotheses) in *SI Appendix*, section 2.A deliver similar messages.

**Fig. 8.**
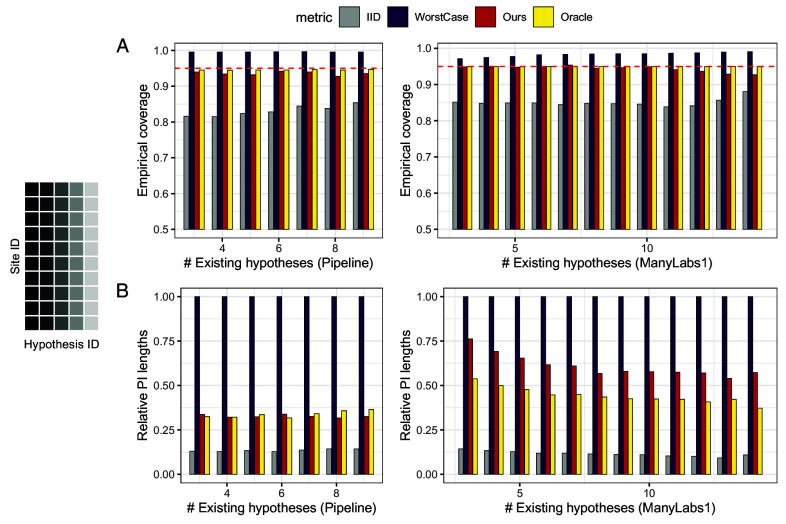
Effect generalization with auxiliary data: We generalize to new hypotheses based on distribution shift measures calibrated from the same sites in other hypotheses. *Left*: Data collection order, where dark color means earlier. Row (*A*): Empirical coverage of prediction intervals built with four methods over 10 random draws of hypotheses ordering, using the Pipeline (*Left*) and ManyLabs 1 (*Right*) data. The red dashed line is the nominal level 0.95. Row (*B*): Average length of prediction intervals over 10 random draws of hypotheses ordering, normalized by the largest average length, using the Pipeline (*Left*) and ManyLabs 1 (*Right*) data.

Fig. 8 reports the coverage and lengths of prediction intervals. For both projects, our procedure achieves coverage close to the nominal level, with prediction intervals that are much smaller than those based on worst-case bounds, and quite close to the oracle method. As before, prediction intervals based on the i.i.d. assumption exhibit undercoverage.

## 5. Robustness under Purposive Sampling

So far, we have focused on collaborative multisite replication studies to understand realistic distribution shifts in the most idealized setting where researchers make best efforts to be consistent, and use a random shift model to capture all unintended discrepancies that are impossible to fully eliminate (22). However, there are many other cases where intended variations of the study population (41, 56) or experimental design (39) can provide valuable insights into the heterogeneity and robustness of treatment effects.

In this section, we extend our insights to other generalization scenarios. We conduct the same set of empirical analyses to a dataset with deliberately chosen diverse populations and explain the observed phenomenon under a hybrid model.

### 5.1. Other Generalization Scenarios.

We begin with several alternative scenarios of generalization and their implications to our analysis and theory. These scenarios deviate from—and could help clarify—the scope of our current model.

As a first example, researchers may choose specific sites that are believed to differ from each other and provide diverse evidence (41, 56, 57). Each site then independently recruits participants and collects data using the same procedure. In this case, one may still expect inevitable random changes (22) that can be captured by ideas similar to our model. However, certain “deterministic” shift is present reflecting the researchers’ decisions. Our empirical analyses can still be carried out for such datasets as long as the variables and parameters are defined consistently across studies, yet the theoretical modeling may not be suitable any more.

As a second example, researchers may explicitly control the distribution of certain observed covariates of the recruited participants. In the most extreme case, the distribution of observed covariates is exactly the same across sites, whereas that of unobserved effect modifiers might differ. It is then impossible to gauge the unobserved shifts without additional information. Instead, one may need worst-case analysis assuming certain degree of distribution shift in the outcomes.

As a third example, researchers may deliberately vary the experimental design (22, 39)—such as the experimental materials, procedures, and treatment assignment mechanisms—to investigate the robustness of a scientific hypothesis. This may lead to inconsistent definitions of variables, parameters, and estimates. Thus, the analysis and theory in this paper may not apply, and we believe new empirical and theoretical frameworks are needed for these settings.

In the remaining of this section, we study a dataset close to the first example where the analyses are still applicable.

### 5.2. Analyzing the KSJ Data.

(41) argues the importance of diverse populations for understanding heterogeneity across studies. To this end, they deliberately chose several online and offline populations that are expected to differ (we take panels from studies 1 to 2, totaling 13 panels for 4 hypotheses; we refer to this as the KSJ data hereafter). While our random shift model also allows for diverse study populations, it cannot address the deterministic process of site selection by the investigators. Nevertheless, as the variables and parameters are consistent across panels, we can still analyze the relationship between covariate and conditional shifts.

**Fig. 9.**
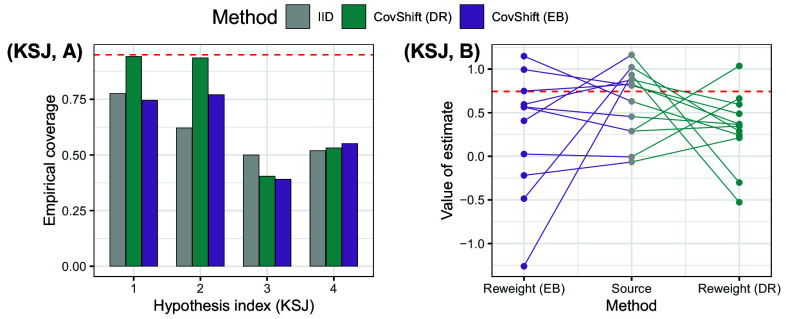
Explanatory role of covariate shift in the KSJ dataset; details are as in Fig. 3. Panel (*KSJ, A*) shows empirical coverage, and Panel (*KSJ, B*) shows the estimates for different procedures.

Identical to the analyses in Section 2.3, we first study effect generalization methods based on the i.i.d. assumption and the covariate shift assumption. Fig. 9 shows the presence of significant distribution shift (IID), and the insufficient explanatory role of covariate shift (CovShift).

**Fig. 10.**
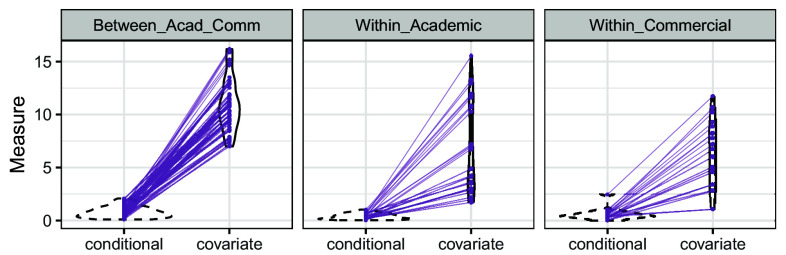
Comparing the strengths of covariate and conditional shifts in various contexts in the KSJ dataset; details are the same as Fig. 4.

We then analyze the covariate and conditional shifts, using the same methods as in Section 3.2. Similar to Fig. 4, we compute $\left({\hat{t}}_{Y|\;X},{\hat{t}}_{X}\right)$ for each pair of panels, and compare their strengths across contexts (“academic”/ “commercial” panels detailed in *SI Appendix*, section 1). In Fig. 10, we observe that the covariate shift measures upper bound the conditional shift measures across various contexts for most site pairs, although the ratio between the conditional and covariate shift measures seems much smaller. When the conditional shifts are larger (comparing across panels), the covariate shifts are also larger.

**Fig. 11.**
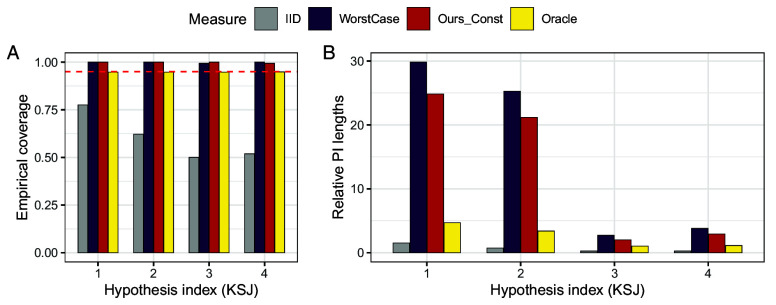
Prediction intervals via constant calibration for effect generalization in the KSJ dataset; details are the same as Fig. 7. Panel (*A*) shows coverage, and panel (*B*) shows the relative lengths of prediction intervals.

**Fig. 12.**
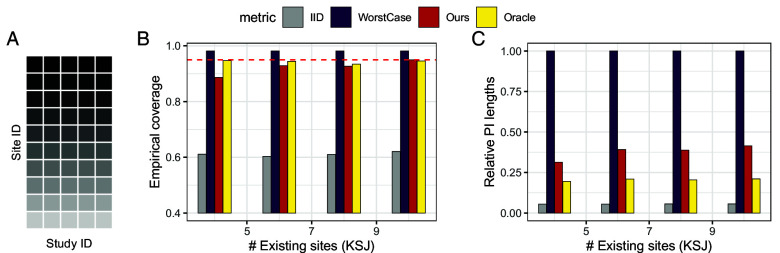
Data-adaptive calibration for effect generalization, where distribution shifts in a subset of existing sites are used to calibrate the bounds for the other sites Panel (*A*). Panel (*B*) reports empirical coverage; Panel (*C*) reports the relative lengths of the prediction intervals.

Finally, following Section 4, we use the predictive role to calibrate prediction intervals for new generalization tasks. The constant calibration (same as Section 4.2.1) is illustrated in Fig. 11. We observe that assuming a constant bound leads to valid coverage; our method is again more efficient than WorstCase, yet is much more conservative than Oracle, again showing the conservativeness of the predictive role. In addition, due to the limited number of hypotheses, we consider a data-adaptive calibration scenario where full observations are available for a random subset of sites, and the new generalization tasks concern the remaining sites. In Fig. 12, when calibrating the bounds $\left(r_{L},r_{U}\right)$ with existing data, our method becomes much more efficient.

To summarize, our analysis of the KSJ data again finds the predictive role of the covariate shift, albeit being conservative. Consequently, constant calibration using the predictive role leads to valid yet conservative uncertainty quantification; with auxiliary data, the data-adaptive calibration approach greatly improves the efficiency while maintaining validity.

### 5.3. Interpretation with Hybrid Shift.

Finally, we provide a high-level discussion on why the conservative predictive role may hold in this purposive sampling scenario. In the KSJ dataset, the distribution shift induced by the choice of researchers can be thought of as deterministic. In addition, there may be numerous random factors in the data collection process which perturb the “ideal” site distributions selected by the researchers in a way similar to our random shift model. Altogether, it would be reasonable to imagine a mixture of deterministic and random distribution shift. In the deterministic part, one would expect the sites/panels to be more diverse in observed variables—since that is what the investigators diversify—than in unobserved variables. On the other hand, the unintended changes in the random shift would lead to perturbations of similar strength to observed and unobserved variables. Combining the two, the estimated covariate shift would be larger than the conditional shift, leading to a conservative predictive role observed here.

## 6. Discussion

In this work, we offer insights on distribution shifts when inferring parameter estimates in a new site based on data from one site and covariates from the new one. By empirical benchmarking in large-scale replication projects, we find significant distribution shifts between sites. However, approaches that only account for shifts of observed covariates—thereby relying on the *explanatory* role of covariate shift—are often insufficient for explaining discrepancies between sites.

Instead of using covariates in an explanatory fashion, we propose to use covariates in a predictive fashion. More precisely, we suggest predicting the strength of the shift of unobserved conditional distribution based on that of observed covariates. We provide empirical evidence based on large-scale replication studies and offer a theoretical justification under a random distribution shift model. In our empirical applications, we show that our proposed prediction intervals maintain the desired coverage even in the presence of unobservable shifts. While these intervals can sometimes be overconservative, they offer a significant improvement over existing approaches. Our method compares favorably to worst-case approaches, which tend to be overly pessimistic.

Our empirical and theoretical findings open up several exciting future avenues. First, real-world scenarios may involve more complex forms of distribution shifts than the one studied in this work (e.g., those discussed in Section 5.1). Empirical understanding and development of estimation procedures for these new models would be a valuable contribution. Second, the nonnegligible conditional shift suggests the importance of collecting data from diverse sources to properly address the “distributional uncertainty”. Toward this goal, our investigation can provide insights for an important methodological challenge: prioritizing data collection. For example, with a partial covariate shift and partial random shift, it may be beneficial to prioritize the collection of covariates most affected by the shift.

## Supplementary Material

Appendix 01 (PDF)

## Data Availability

Previously published data were used for this work (16, 17). All other data are included in the manuscript and/or *SI Appendix*.

## Footnotes

^*^Note that not all sites examine all hypotheses.

^†^We use prediction intervals rather than the conventional CIs because we only have access to target population estimates (instead of the underlying parameters) for rigorous evaluation purposes.

^‡^That is, for any function $\phi \left(X,U\right)\in L^{2}\left(P\right)$, it holds that $\lim_{M\to \infty }E_{P}\left[{\left(\phi \left(X,U\right)-\phi_{M}\left(X,U\right)\right)}^{2}\right]=0$, where $\phi_{M}=\sum_{m=1}^{M}1_{C_{m}^{\left(M\right)}}E_{P}\left[\phi \left(X,U\right)|C_{m}^{\left(M\right)}\right]$. This can be achieved relatively easily, e.g. for a continuous random variable X∈R one can set $C_{m}$ as intervals whose endpoints are the (m−1)/M-th and m/M-th quantiles of X under P.

^§^We again create prediction intervals for easier evaluation based on target estimates (instead of the underlying parameters).

^¶^Such computation of ρ is unavailable in a real generalization task, and the lower/upper bounds for the parameter are anticonservative for prediction interval.
