# Supplementary material for: Beyond reweighting: On the predictive role of covariate shift in effect generalization
Source: Proc Natl Acad Sci U S A. 2025 Nov 3;122(45):e2427181122. doi: 10.1073/pnas.2427181122 (PMC12625858; doi:10.1073/pnas.2427181122)
Supplement: Supplementary file 1 — Appendix 01 (PDF) [file pnas.2427181122.sapp.pdf]

Supporting Information for

Beyond Reweighting: On the Predictive Role of Covariate Shift in Effect Generalization

Ying Jin, Naoki Egami and Dominik Rothenhäusler

Dominik Rothenhäusler.
E-mail: rdominik@stanford.edu

This PDF file includes:

Figs. S1 to S14
Tables S1 to S7

1. Details of datasets and data pre-processing

A. Pre-processing for Pipeline project. The raw datasets for the Pipeline project can be found in the OSF repository https://osf.io/q25xa/. The detailed data pre-processing script can be found in the folder `Pipeline` in the GitHub respository https://github.com/ying531/awesome-replicability-data.

We follow the data processing scripts (in the folder "SPSS Syntax files") provided in the OSF repository to compute the response variables, encode the treatment indicators, and extract the covariates including age, gender, country of birth, language, ethnicity, parent education, and family incomes. When running the analysis, we additionally process the data for each site as follows: covariates with all N/A values are excluded; otherwise, the missing observations are imputed by the site median. Since entropy balancing enforces positive weights, when running the EB-based methods, we also exclude covariates whose sample average in the target dataset falls outside the support in the source dataset.

B. Pre-processing for ManyLabs1 project. The raw datasets for the ManyLabs1 project can be found in the OSF repository https://osf.io/wx7ck/. The detailed data processing script can be found in the folder `ManyLabs1` in the GitHub respository https://github.com/ying531/awesome-replicability-data.

We follow the data processing scripts `Syntax.Manylabs.sps` in the OSF repository to encode the responses (`dv`) and treatment indicators (`iv`), and extract the covariates including gender, age, race, ethnicity, nationality, native language, religion, and ideology.

C. Pre-processing for KSJ dataset. The raw dataset for the KSJ dataset can be found in the OSF repository https://osf.io/7kqg9/, where we take the 13 panels in study 1 and 2, making only minor changes to the variable names basedon how they are labeled in the original datasets. The detailed data processing script can be found in the folder `KSJ` in the GitHub repository https://github.com/ying531/awesome-replicability-data.

D. Reproduction code. The code for reproducing the analysis is available at https://github.com/ying531/predictive-shift. For easier reproduction, we also include analyses results (such as computed distribution shift measures and constructed KL-based bounds which can be costly to run) ready for producing the figures in the main text.

E. Dataset information. Table S1 lists the data indices and data collection sites for the Pipeline project from the Open Science Framework (OSF) repository. Table S2 summarizes the information for each of the 10 hypothesis studied in the Pipeline project, including the name, test statistic and formula, number of sites conducting experiments for testing this hypothesis, and total sample sizes N recruited in these sites.

Table S3 lists the data collection sites in the ManyLabs1 project. Table S4 summarizes the information for each of the 15 hypotheses studied in the ManyLabs1 dataset, including the hypothesis, estimator, formula (for processed data), number of sites conducting experiments for the hypothesis, and total sample sizes N.

Table S5 summarizes the information of the 4 hypotheses studied in the KSJ dataset, including the hypothesis, estimator, formula (for processed data), number of sites conducting experiments for the hypothesis, and total sample sizes N. Table S6 lists the 13 panels in our analysis and their characteristics provided in (41).

ID	Hypothesis	Estimator	Formula	Number of Sites	N
1	Bigot–misanthrope	t-test	`bigot_personjudge ~ condition`	12	2861
2	Cold-hearted prosociality	Paired t-test	`tdiff ~ 1`	12	2806
3	Bad tipper	t-test	`tipper_personjudg ~ condition`	16	3658
4	Belief–act inconsistency	t-test	`beliefact_mrlblmw_rec ~ condition13`	13	3006
5	Moral inversion	t-test	`moralgood ~ condition`	14	3076
6	Moral cliff	Paired t-test	`diff ~ 1`	15	3300
7	Intuitive economics	t-test	`yz ~ condition`	15	3164
8	Burn-in-hell	Paired t-test	`tdiff ~ 1`	15	3176
9	Presumption of guilt	t-test	`companyevaluation ~ condition`	17	3806
10	Higher standard	t-test	`standard_evalu_7items ~ condition`	11	2692

Table S2. Estimator, number of sites and total sample size N for each hypothesis in the Pipeline project.

New Index	Raw ID	PI, Institution
1	0	Original Study data collection
2	1	Aaron Sackett, University of St. Thomas
3	2	Alexandra Mislin, American University
4	4	David Tannenbaum, University of Chicago
5	5	Daniel Storage, University of Illinois at Urbana-Champaign
6	6	Adam Hahn, University of Cologne
7	7	Nicole Legate, Illinois Institute of Technology
8	8	INSEAD Sorbonne Lab
9	9	Victoria Brescoll, Yale University
10	10	Felix Cheung, Michigan State University/University of Hong Kong
11	11	Fiery Cushman, Harvard University
12	12	Jay Van Bavel, New York University
13	13	Tatiana Sokolova, HEC Paris and University of Michigan
14	15	Jesse Graham, University of Southern California
15	16	Anne-Laure Sellier, HEC Paris
16	17	Eli Awtrey, University of Washington
17	18	Jennifer Jordan, University of Groningen
18	19	Sapna Cheryan, University of Washington
19	20	Xiaomin Sun, Beijing Normal University
20	21	Yoel Inbar, University of Toronto
21	22	Wendy Bedwell, University of South Florida
22	24	Deanna Kennedy, University of Washington Bothell
23	25	Matt Motyl, University of Illinois at Chicago
24	26	Erik Cheries, University of Massachusetts Amherst
25	27	Additional INSEAD-Sorbonne lab data for Study 1
26	141	Dan Molden, Packet 1 for Study 7
27	142	Dan Molden, Packet 2 for Study 4 and Study 8
28	311	UCI Psychology Students
29	312	UCI Business Students

Table S1. List of new index, raw site ID in the dataset, and contributing PI and site institutions in the Pipeline project dataset, taken from the Open Science Framework project repository (16).

New Index	Raw Site ID	Institution, Location
1	Abington	Penn State Abington, Abington, PA
2	Brasilia	University of Brasilia, Brasilia, Brazil
3	Charles	Charles University, Prague, Czech Republic
4	Conncoll	Connecticut College, New London, CT
5	CSUN	California State University, Northridge, LA, CA
6	Help	HELP University, Malaysia
7	Ithaca	Ithaca College, Ithaca, NY
8	JMU	James Madison University, Harrisonburg, VA
9	KU	Koç University, Istanbul, Turkey
10	Laurier	Wilfrid Laurier University, Waterloo, Ontario, Canada
11	LSE	London School of Economics and Political Science, London, UK
12	Luc	Loyola University Chicago, Chicago, IL
13	McDaniel	McDaniel College, Westminster, MD
14	MSVU	Mount Saint Vincent University, Halifax, Nova Scotia, Canada
15	MTURK	Amazon Mechanical Turk (US workers only)
16	OSU	Ohio State University, Columbus, OH
17	Oxy	Occidental College, LA, CA
18	PI	Project Implicit Volunteers (US citizens/residents only)
19	PSU	Penn State University, University Park, PA
20	QCCUNY	Queens College, City University of New York, NY
21	QCCUNY2	Queens College, City University of New York, NY
22	SDSU	SDSU, San Diego, CA
23	SWPS	University of Social Sciences and Humanities Campus Sopot, Sopot, Poland
24	SWPSON	Volunteers visiting www.badania.net
25	TAMU	Texas A&M University, College Station, TX
26	TAMUC	Texas A&M University-Commerce, Commerce, TX
27	TAMUON	Texas A&M University, College Station, TX (Online participants)
28	Tilburg	Tilburg University, Tilburg, Netherlands
29	UFL	University of Florida, Gainesville, FL
30	UNIPD	University of Padua, Padua, Italy
31	UVA	University of Virginia, Charlottesville, VA
32	VCU	VCU, Richmond, VA
33	Wisc	University of Wisconsin-Madison, Madison, WI
34	WKU	Western Kentucky University, Bowling Green, KY
35	WL	Washington & Lee University, Lexington, VA
36	WPI	Worcester Polytechnic Institute, Worcester, MA

Table S3. List of new index, raw site ID in the dataset, and contributing PI and site institutions in the ManyLabs 1 dataset, taken from the original paper (17).

ID	Hypothesis	Estimator	Formula	Number of Sites	N
1	Allowedforbidden	t-test	`dv ~ iv`	36	6292
2	Anchoring1	t-test	`dv ~ iv`	36	5362
3	Anchoring2	t-test	`dv ~ iv`	36	5284
4	Anchoring3	t-test	`dv ~ iv`	36	5627
5	Anchoring4	t-test	`dv ~ iv`	36	5609
6	Contact	t-test	`dv ~ iv`	36	6336
7	Flag	t-test	`dv ~ iv`	36	6251
8	Gainloss	t-test	`dv ~ iv`	36	6271
9	Gambfal	t-test	`dv ~ iv`	36	5942
10	Iat	t-test	`dv ~ iv`	36	5851
11	Money	t-test	`dv ~ iv`	36	6333
12	Quote	t-test	`dv ~ iv`	36	6325
13	Reciprocity	t-test	`dv ~ iv`	36	6276
14	Scales	t-test	`dv ~ iv`	36	5899
15	Sunk	t-test	`dv ~ iv`	36	6330

Table S4. Estimator, number of sites and total sample size N for each hypothesis (indices and variable names in cleaned data and this paper) for ManyLabs 1 data.

ID	Hypothesis	Estimator	Formula	Number of Panels	N
1	sunk-cost	t-test	`numSunkCost` $\sim$ `SunkCondition`	13	4909
2	less-is-better	t-test	`numLessMore` $\sim$ `LessMoreCondition`	13	4909
3	framing	t-test	`numDisease` $\sim$ `DiseaseCondition`	13	4909
4	default	t-test	`numDefault` $\sim$ `DefaultCondition`	13	4909

Table S5. Estimator, number of panels and total sample size N for each hypothesis in the KSJ dataset.

Raw Site ID	Sample size	Academic or commercial	Source study in KSJ
Blended	564	Commercial	Study 1A
BrandedResearch	534	Commercial	Study 1A
CloudResearch	491	Academic	Study 1A
MTurk	302	Academic	Study 1A
MTurk_2	311	Academic	Study 2
Prodege	330	Commercial	Study 1A
Prolific	328	Academic	Study 1A
Prolific_2	331	Academic	Study 2
Prolific_UK_Rep	324	Academic	Study 1B
Prolific_US_Rep	315	Academic	Study 1B
Students	402	Academic	Study 1B
TapResearch	329	Commercial	Study 1A
inBrain.ai	348	Commercial	Study 1A

Table S6. List of panel name, sample size (after removing missing values in key variables), and panel characteristics in the KSJ dataset.

2. Estimation details

In this section, we detail the estimation procedures for all the analyses in this paper. Appendix 2.A recalls important notations. Appendix B describes the analysis for the explanatory role in Section 1.C in the main text. Appendix 2.C details the estimation for our distribution shift measures in Section 2 in the main text. Finally, Appendix 2.D details our estimation and evaluation procedures for effect generalization in Section 3 in the main text.

A. Notations. We begin by revisiting some notations. A hypothesis k is replicated by sites $j \in \{1, \dots, N_k\}$, each observing a dataset $\mathcal{D}_j^{(k)} = \{X_i^{(j,k)}, T_i^{(j,k)}, Y_i^{(j,k)}\}_{i=1}^{n_j^{(k)}}$, where X_i is the covariates, $T_i \in \{0,1\}$ is the binary treatment, and Y_i is the outcome(s). For each hypothesis k, the estimate for site j is $\hat{\theta}_j^{(k)} = \theta^{(k)}(\mathcal{D}_j^{(k)})$, where $\theta^{(k)}$ is the same functional that represents the analysis procedure applied to all sites (as listed in Tables S2 and S4). Here, $\hat{\theta}_j^{(k)}$ estimates the population parameter $\theta_j^{(k)} = \theta^{(k)}(P_j^{(k)})$, where $P_j^{(k)}$ is the underlying distribution from which $\mathcal{D}_j^{(k)}$ is drawn. We assume access to a function $\phi^{(k)}(\cdot)$ such that

$$\hat{\theta}_j^{(k)} = \frac{1}{n_j^{(k)}} \sum_{i=1}^{n_j^{(k)}} \phi^{(k)}\big(X_i^{(j,k)}, Y_i^{(j,k)}, T_i^{(j,k)}\big).$$

B. Estimation for the explanatory role. In this part, we detail how the prediction intervals for IID, CovShift (DR) and CovShift (EB) are constructed and evaluated in Section 1.C in the main text. The sites are denoted as $i, j \in \{1, \dots, N\}$, where site i is the "original" site with full observations, and site j is the "target" site we want to generalize the effects to.

Estimation for IID. For any site pair (i,j) for a hypothesis k, we assume access to a consistent variance estimator $(\hat{\sigma}_i^{(k)})^2$ for $\hat{\theta}_i^{(k)}$, such that

$$\sqrt{n_i^{(k)}} \cdot \frac{\hat{\theta}_i^{(k)} - \theta_i^{(k)}}{\hat{\sigma}_i^{(k)}} \xrightarrow{d} N(0,1).$$

Note that $\hat{\theta}_i$ and $\hat{\sigma}_i$ can be computed using full observations $\mathcal{D}_i^{(k)}$ from the "original" site i. It is straightforward to construct these estimators for the t-tests and paired t-tests considered in this work, and we note that $\hat{\sigma}_i^{(k)} = \hat{\sigma}_j^{(k)} + o_P(1)$ for any $i \neq j$ if the i.i.d. assumption holds. For the `IID` method, we construct a prediction interval based on site i for $\hat{\theta}_j^{(k)}$ via

$$\hat{C}_{i \to j}^{\mathrm{IID},(k)} = \hat{\theta}_i^{(k)} \pm q_{1-\alpha/2} \cdot \hat{\sigma}_i^{(k)} \cdot \left(\sqrt{1/n_i^{(k)} + 1/n_j^{(k)}}\right), \qquad [1]$$

where $q_{1-\alpha/2}$ is the $(1-\alpha/2)$-th quantile of a standard normal distribution. Under the i.i.d. assumption that $P_i^{(k)} = P_j^{(k)}$, we know that

$$\mathbb{P}\left(\hat{\theta}_j^{(k)} \in \hat{C}_{i\to j}^{\mathrm{IID},(k)}\right) \to 1-\alpha.$$

For evaluation, we will use full observations from the target site. Each grey bar in (P,a) and (M,a) of Figure 3 is computed via

$$\hat{\mathrm{Cov}}_k^{\mathrm{IID}} := \frac{1}{N_k(N_k-1)} \sum_{i=1}^{N_k} \sum_{j\neq i} \mathbb{1}\left\{\hat{\theta}_j^{(k)} \in \hat{C}_{i\to j}^{\mathrm{IID},(k)}\right\}.$$

Thus, if the i.i.d. assumption holds, we will expect $\hat{\mathrm{Cov}}_k^{\mathrm{IID}} \approx 1-\alpha$.

Estimation for CovShift (DR). For any site pair (i,j) in a hypothesis k, we first describe how to construct a point estimate for generalization via reweighting. We denote the estimator as $\hat{\theta}_{i\to j}^{(k)}$ when generalizing from site i with full observations to site j with only covariate information. We will employ cross-fitting (**?**) to allow the use of flexible machine learning algorithms such as random forests in estimating the covariate shift weights and conditional mean functions.

First, we randomly split the data $\mathcal{D}_i^{(k)}$ and covariates in $\mathcal{D}_j^{(k)}$ into two equally-sized halves each. We use one half of data to estimate the covariate shift function $\mathrm{d}P_{j,X}^{(k)}/\mathrm{d}P_{i,X}^{(k)}(x)$ via $\hat{w}(x)$, and the conditional mean function $\varphi(x) := \mathbb{E}[\phi^{(k)}(X,Y,T)\,|\,X = x]$ via $\hat{\varphi}(x)$. These functions will be applied to the other fold of data, and construct the reweighted estimator

$$\hat{\theta}_{i\to j}^{(k)} = \frac{1}{n_i^{(k)}} \sum_{\ell} \hat{w}(X_\ell^{(i,k)}) \cdot \left\{\phi^{(k)}(X_\ell^{(i,k)}, Y_\ell^{(i,k)}, T_\ell^{(i,k)}) - \hat{\varphi}(X_\ell^{(i,k)})\right\} + \frac{1}{n_j^{(k)}} \sum_{\ell=1}^{n_j^{(k)}} \hat{\varphi}(X_\ell^{(j,k)}). \quad [2]$$

Following (51), if the covariate shift condition holds, one can show that for the t-test and paired t-test considered in this work, as long as $\hat{w}$ and $\hat{\varphi}$ converge to the true covariate shift weight function and the true conditional mean function with a rate of $o_P((n_i^{(k)})^{-1/4})$, it holds that

$$\frac{\hat{\theta}_j^{(k)} - \hat{\theta}_{i\to j}^{(k)}}{\hat{\sigma}_{i\to j}^{(k),\mathrm{CovShift}}} \stackrel{d}{\to} N(0,1),$$

where $\hat{\sigma}_{i\to j}^{(k),\mathrm{CovShift}}$ is any consistent estimator for $\sigma_{i\to j}^{(k),\mathrm{CovShift}}$, and

$$(\sigma_{i\to j}^{(k),\mathrm{CovShift}})^2 = \frac{\mathbb{E}_i^{(k)}[w(X)^2 \cdot (\phi^{(k)}(X,Y,T) - \varphi^{(k)}(X))^2]}{n_i^{(k)}} + \frac{\mathbb{E}_i^{(k)}[w(X) \cdot (\phi^{(k)}(X,Y,T) - \varphi^{(k)}(X))^2]}{n_j^{(k)}}.$$

As such, we construct the prediction interval for CovShift (DR) via

$$\hat{C}_{i\to j}^{\mathrm{CovShift},(k)} = \hat{\theta}_{i\to j}^{(k)} \pm q_{1-\alpha/2} \cdot \hat{\sigma}_{i\to j}^{(k),\mathrm{CovShift}},$$

where $\hat{\sigma}_{i\to j}^{(k),\mathrm{CovShift}}$ is constructed by plugging in $\hat{w}$ and $\hat{\varphi}$ into the definition of $\sigma_{i\to j}^{(k),\mathrm{CovShift}}$. Based on the arguments above, assuming covariate shift, under standard assumptions above, we would have

$$\mathbb{P}\left(\hat{\theta}_j^{(k)} \in \hat{C}_{i\to j}^{\mathrm{CovShift},(k)}\right) \to 1-\alpha.$$

For evaluation, we will use full observations from the target site. Each green bar in (P,a) and (M,a) of Figure 3 is computed via

$$\hat{\mathrm{Cov}}_k^{\mathrm{CovShift}} := \frac{1}{N_k(N_k-1)} \sum_{i=1}^{N_k} \sum_{j\neq i} \mathbb{1}\left\{\hat{\theta}_j^{(k)} \in \hat{C}_{i\to j}^{\mathrm{CovShift},(k)}\right\}.$$

If the covariate shift assumption holds, we expect $\hat{\mathrm{Cov}}_k^{\mathrm{CovShift}} \approx 1-\alpha$ under standard regularity conditions.

Estimation for CovShift (EB). The idea for constructing the point estimate for CovShift (EB) is similar to CovShift (DR), with the only exception that we obtain the weights $\hat{w}_\ell^{(i,k)}$ are obtained by entropy balancing (28) following the procedure in (14), while $\hat{\sigma}_{i\to j}^{(k),\mathrm{CovShift}}$ is obtained in the same way as in CovShift (DR). We then construct the point estimate

$$\hat{\theta}_{i\to j}^{(k)} = \frac{1}{n_i^{(k)}} \sum_{\ell} \hat{w}_\ell^{(i,k)} \cdot \phi^{(k)}(X_\ell^{(i,k)}, Y_\ell^{(i,k)}, T_\ell^{(i,k)}) \quad [3]$$

and prediction interval

$$\hat{C}_{i\to j}^{\mathrm{CovShift},(k)} = \hat{\theta}_{i\to j}^{(k)} \pm q_{1-\alpha/2} \cdot \hat{\sigma}_{i\to j}^{(k),\mathrm{CovShift}}.$$

Following (14), assuming covariate shift, if the weight is a logistic function of the covariates or if $\varphi(x)$ is a linear function of the covariates, we would have

$$\mathbb{P}\Big(\hat{\theta}_j^{(k)} \in \hat{C}_{i\to j}^{\text{CovShift},(k)}\Big) \to 1-\alpha.$$

For evaluation, we will use full observations from the target site. For evaluation, each purple bar in (P,a) and (M,a) of Figure 3 is computed via

$$\hat{\text{Cov}}_k^{\text{CovShift}} := \frac{1}{N_k(N_k-1)} \sum_{i=1}^{N_k} \sum_{j\neq i} \mathbb{1}\left\{\hat{\theta}_j^{(k)} \in \hat{C}_{i\to j}^{\text{CovShift},(k)}\right\}$$

using the prediction intervals for CovShift (EB). Thus, if the covariate shift assumption holds, we will expect $\hat{\text{Cov}}_k^{\text{CovShift}} \approx 1-\alpha$ under the stated linear assumptions which are standard in the balancing literature. We note that CovShift (EB) is more stable than CovShift (DR) for small-to-moderate sample sizes, as is the case for the datasets analyzed in this work.

C. Estimation for distribution shift measures. We then proceed to detail the estimation procedure for our new distribution shift measures. To begin with, we note the following decomposition by (14), which measures the contributions of distribution shifts (on the super-population level) to effect discrepancy:

$$\theta(Q) - \theta(P) = \underbrace{\theta(Q) - \theta(Q_X \times P_{Y|X})}_{\text{Contribution of conditional shift}} + \underbrace{\theta(Q_X \times P_{Y|X}) - \theta(P)}_{\text{Contribution of covariate shift}} \qquad [4]$$

where $\theta(\cdot)$ is the functional for the parameter of interest, P is the source distribution, Q is the target distribution, and $Q_X \times P_{Y|X}$ is the reweighted distribution. Note that the contribution of conditional shift will be zero under the covariate shift assumption (Definition 1). In multi-site replication studies, for generalizing estimates for a hypothesis k from site i to site j, we will take $\theta = \theta^{(k)}$, $P = P_i^{(k)}$, and $Q = P_j^{(k)}$.

Computing the conditional shift measure. Following [4], we recall our definitions of the population-level conditional shift measure (for generalizing from P to Q) in Section 2.A in the main text, denoted as

$$t_{Y|X} := \frac{\Delta_{Y|X}}{s_{Y|X}}, \quad \Delta_{Y|X} = \theta(Q) - \theta(Q_X \times P_{Y|X}), \quad s_{Y|X}^2 = \text{Var}_P\left(\phi(X,Y,T) - \mathbb{E}_P[\phi(X,Y,T) \,|\, X]\right),$$

where the contributions of the conditional shift is rescaled by the standard deviation of its influence function to ensure scale invariance.

Following the notations in the preceding subsection, we compute the conditional shift measure from site i to site j in hypothesis k via the following formula:

$$\hat{t}_{Y|X}^{i\to j,(k)} = \frac{\hat{\Delta}_{Y|X}^{i\to j,(k)}}{\hat{s}_{Y|X}^{i\to j,(k)}} := \frac{\hat{\theta}_j^{(k)} - \hat{\theta}_{i\to j}^{(k)}}{\hat{s}_{Y|X}^{i\to j,(k)}} \qquad [5]$$

where $\hat{\theta}_j^{(k)}$ is the target estimator for $\theta(Q)$, $\hat{\theta}_{i\to j}^{(k)}$ is the doubly robust estimator [2] or the entropy balancing estimator [3] in the previous part, so that $\hat{\Delta}_{Y|X}$ is an estimator for the contribution of conditional shift. In addition, $\hat{s}_{Y|X}^{i\to j,(k)}$ is a consistent estimator for $\text{Var}_P\left(\phi(X,Y,T) - \mathbb{E}_P[\phi(X,Y,T) \,|\, X]\right)^{1/2}$, which we detail in Appendix 5.C and introduce its fast convergence properties.

Computing the covariate shift measure. Finally, we compute the "stabilizes" covariate shift measure as mentioned in the main text. Namely, supposing there are L covariates $\{X_\ell\}_{\ell=1}^L$, we compute

$$\hat{t}_X^{i\to j,(k)} := \sqrt{\frac{1}{L}\sum_{\ell=1}^L \left(\frac{\hat{\mathbb{E}}_Q[X_\ell] - \hat{\mathbb{E}}_P[X_\ell]}{\hat{\sigma}_P(X_\ell)}\right)^2}, \qquad [6]$$

where $\hat{\sigma}_P(X_\ell)$ is the empirical standard deviation of X_ℓ in the source dataset. Note that $\hat{t}_X^{i\to j,(k)}$ is pivotal as $n_i^{(k)}, n_j^{(k)} \to \infty$ under the i.i.d. assumption.

Computing the ratios. After computing the two measures $\hat{t}_{Y|X}^{i\to j,(k)}$ and $\hat{t}_X^{i\to j,(k)}$, we simply measure their relative strengths by the ratio

$$\hat{r}_{i\to j}^{(k)} = \hat{t}_{Y|X}^{i\to j,(k)} / \hat{t}_X^{i\to j,(k)}.$$

Alternative definitions of distribution shift measures will be explored in Appendix 4.4, yet we find they either (i) are scale-dependent (hence interpretation is sensitive the definition of the parameter functional $\theta(\cdot)$), or (ii) lead to unstable performance in estimation and effect generalization.

Idea for effect generalization based on distribution shift measures. Finally, we recall the high-level idea of effect generalization based on our distribution shift measures. If the distribution of the ratio $\hat{r}^{i\to j,(k)}$ (which depends on both sampling uncertainty and distribution shifts) can be characterized, so that one can find upper and lower bounds L and U (either by asymptotic distribution or data-adaptive calibration) such that (approximately)

$$\mathbb{P}\Big(L \le \hat{r}^{(k)}_{i\to j} \le U\Big) \ge 1-\alpha, \qquad [7]$$

then, inverting this fact would give a prediction interval for $\hat{\theta}^{(k)}_j$, which is

$$\hat{C}^{(k)}_{i\to j} = \Big[\hat{\theta}^{(k)}_{i\to j} + L\cdot \hat{t}_X^{i\to j,(k)}\cdot \hat{s}_X^{i\to j,(k)},\ \hat{\theta}^{(k)}_{i\to j} + U\cdot \hat{t}_X^{i\to j,(k)}\cdot \hat{s}_X^{i\to j,(k)}\Big].$$

Above, except for L and U, all quantities can be estimated with full observations from site i and covariates from site j. Next, we will detail how L and U are calibrated in Section 3 in the main text.

D. Estimation for effect generalization. In this part, we detail our estimation and evaluation procedures for effect generalization in Section 3 in the main text. We first introduce the IID method and the Oracle method evaluated in both Figure 7 and Figure 8. Then, we introduce WorstCase and Ours methods for constant calibration and adaptive calibration in the two figures, respectively.

IID method. With the i.i.d. assumption, we construct prediction intervals as [1] for generalizing from site i to site j for hypothesis k. That is, we use no covariate information in the sites, and the empirical coverage of the IID method is mainly plotted for reference. For coverage and lengths, we average over all site pairs for a given hypothesis in all scenarios.

Oracle method. This method uses all site pairs to calibrate the range of $\hat{r}^{i\to j,(k)}$, namely, we compute

$$r_L^{\mathrm{Orc},(k)} := \mathrm{Quantile}\Big(\alpha/2;\ \big\{\hat{r}^{(k)}_{i\to j}\big\}_{i\ne j}\Big),\quad r_U^{\mathrm{Orc},(k)} := \mathrm{Quantile}\Big(1-\alpha/2;\ \big\{\hat{r}^{(k)}_{i\to j}\big\}_{i\ne j}\Big)$$

for the bounds r_L and r_U in [7]. As its name suggests, it is the ideal prediction interval when we have perfect knowledge of how distribution shifts between all sites for a hypothesis. Note that this approach uses much more information than available in a real generalization task, and is hence evaluated just for reference. For coverage and lengths, we average over all site pairs for a given hypothesis in all scenarios.

D.1. Constant calibration.

Our method (constant calibration). We take constants $L=-1$ and $U=1$ in [7], i.e., we believe that the conditional shift is upper bounded by the covariate shift. This leads to the prediction interval

$$\hat{C}^{\mathrm{Ours},(k)}_{i\to j} = \Big[\hat{\theta}^{(k)}_{i\to j} - \hat{t}_X^{i\to j,(k)}\cdot \hat{s}_X^{i\to j,(k)},\ \hat{\theta}^{(k)}_{i\to j} + \hat{t}_X^{i\to j,(k)}\cdot \hat{s}_X^{i\to j,(k)}\Big],$$

which is computable in a real generalization task with $\mathcal{D}^{(k)}_i$ and covariates in $\mathcal{D}^{(k)}_j$. The barplots in Figure 8 show the empirical coverage

$$\frac{1}{N_k(N_k-1)}\sum_{i\ne j}\mathbb{1}\Big\{\hat{\theta}^{(k)}_j \in \hat{C}^{\mathrm{Ours},(k)}_{i\to j}\Big\}$$

and average lengths

$$\frac{1}{N_k(N_k-1)}\sum_{i\ne j}\Big|\hat{C}^{\mathrm{Ours},(k)}_{i\to j}\Big|$$

after normalization by the largest average length for each hypothesis k.

Worst-case method. We also evaluate the performance of worst-case bounds on the conditional shift, calibrated with data at hand. These worst-case bounds estimate the range of target parameters under the constraint that the unknown conditional shift is bounded in a KL-divergence ball.

Before we introduce our approach, we first remark two aspects about this approach:

1. Rigorously speaking, this is not a feasible generalization approach since we need full observations from all sites (especially the outcomes from the target site) to calibrate the KL bound $\hat{\mathrm{KL}}^{(k)}_{\mathrm{upp}}$, which is typically not available in a real generalization task. As such, we mainly use it for reference.

2. There are several approximations in this approach, since the estimation uncertainty in $\hat{\mathrm{KL}}^{(k)}_{\mathrm{upp}}$ is not accounted for, and it usually needs to account for larger uncertainty to cover the actual estimator than the underlying parameter. Thus, the intervals we obtain here can be viewed as underestimating the actual uncertainty, and a rigorous approach would construct even wider intervals.

Specifically, let P be the source distribution and Q be the target distribution. The strength of conditional shift can be characterized by the KL divergence between the reweighted distribution $Q_X \times P_{Y|X}$ and the target distribution Q, i.e.,

$$\begin{aligned}\mathrm{KL}\big(Q \,\|\, Q_X \times P_{Y|X}\big) &= \mathbb{E}_{Q_X \times P_{Y|X}}\left[\frac{\mathrm{d}Q}{\mathrm{d}(Q_X \times P_{Y|X})}(X,Y) \cdot \log \frac{\mathrm{d}Q}{\mathrm{d}(Q_X \times P_{Y|X})}(X,Y)\right] \\ &= \mathbb{E}_{Q_X \times P_{Y|X}}\left[\frac{\mathrm{d}Q_{Y|X}}{\mathrm{d}P_{Y|X}}(X,Y) \cdot \log \frac{\mathrm{d}Q_{Y|X}}{\mathrm{d}P_{Y|X}}(X,Y)\right].\end{aligned}$$

To estimate this quantity, we first use a classification model to estimate the joint density ratio $\mathrm{d}Q_{X,Y}/\mathrm{d}P_{X,Y}(x,y)$ via $\hat{w}_{X,Y}(\cdot)$, and then the covariate density ratio $\mathrm{d}Q_X/\mathrm{d}P_X(x)$ via $\hat{w}_X(\cdot)$. Then, we estimate the conditional density ratio $\mathrm{d}Q_{Y|X}/\mathrm{d}P_{Y|X}(x,y)$ via $\hat{w}_{X,Y}(x,y)/\hat{w}_X(x)$, and plug in the definition to obtain an estimator for the KL-divergence, denoted as $\hat{\mathrm{KL}}_{i\to j}^{(k)}$ when taking $P = P_i^{(k)}$ and $Q = P_j^{(k)}$.

After obtaining $\hat{\mathrm{KL}}_{i\to j}^{(k)}$ for all pairs of studies, we calibrate an upper bound for the conditional KL-divergence for any given hypothesis k via

$$\hat{\mathrm{KL}}_{\mathrm{upp}}^{(k)} := \mathrm{Quantile}\Big(0.99;\ \{\hat{\mathrm{KL}}_{i\to j}^{(k)}\}_{i\neq j}\Big),$$

where we take the 0.99 quantile to avoid outliers. Then, we compute upper and lower bounds for the parameters $\theta(P_j^{(k)})$ by solving the following optimization program:

$$\begin{aligned}\text{Maximize/minimize}\quad & \theta(\bar{Q}) \\ \text{Subject to}\quad & \mathrm{KL}\big(\bar{Q} \,\|\, Q_X \times P_{Y|X}\big) \leq \hat{\mathrm{KL}}_{\mathrm{upp}}^{(k)}.\end{aligned}$$

Algorithms for solving the above program with data are standard in the literature; see, e.g., (**?**). We then use the maximized and minimized objective as upper and lower bounds for the target estimator, giving rise to the prediction interval

$$\hat{C}_{i\to j}^{\mathrm{KL},(k)} := \Big[\hat{L}_{i\to j}^{\mathrm{KL},(k)},\ \hat{U}_{i\to j}^{\mathrm{KL},(k)}\Big].$$

The barplots in Figure 8 show the empirical coverage

$$\frac{1}{N_k(N_k-1)}\sum_{i\neq j}\mathbb{1}\left\{\hat{\theta}_j^{(k)} \in \hat{C}_{i\to j}^{\mathrm{KL},(k)}\right\}$$

and average lengths

$$\frac{1}{N_k(N_k-1)}\sum_{i\neq j}\left|\hat{C}_{i\to j}^{\mathrm{KL},(k)}\right|$$

after normalization for each hypothesis k.

D.2. Data-adaptive calibration. Data-adaptive calibration uses separate datasets, which we assume to be available at a generalization task, to calibrate the strength of distribution shift. We will follow the notations in the preceding part.

Ours (data-adaptive calibration). In Figure 8, we assume that data for hypothesis $k_1,\dots,k_t$ are available when we want to generalize between sites for a new hypothesis k_{t+1}. Thus, we calibrate the lower and upper bounds in [7] at step t by

$$r_L^{\mathrm{Ours},(t)} := \mathrm{Quantile}\Big(\alpha/2;\ \big\{\hat{r}_{i\to j}^{(k_s)}\big\}_{i\neq j, s\leq t}\Big),\quad r_U^{\mathrm{Ours},(t)} := \mathrm{Quantile}\Big(1-\alpha/2;\ \big\{\hat{r}_{i\to j}^{(k_s)}\big\}_{i\neq j, s\leq t}\Big).$$

The idea is that if the distribution of $\hat{r}_{i\to j}^{(k)}$ is "pivotal" across hypothesis, using data for other hypotheses (other outcomes) to calibrate new hypotheses will lead to reliable coverage. We then construct prediction intervals for sites in new hypotheses k_s, $s > t$ by

$$\hat{C}_{i\to j}^{(k_s)} = \Big[\hat{\theta}_{i\to j}^{(k_s)} + r_L^{\mathrm{Ours},(t)} \cdot \hat{t}_X^{i\to j,(k_s)} \cdot \hat{s}_X^{i\to j,(k_s)},\ \hat{\theta}_{i\to j}^{(k_s)} + r_U^{\mathrm{Ours},(t)} \cdot \hat{t}_X^{i\to j,(k_s)} \cdot \hat{s}_X^{i\to j,(k_s)}\Big].$$

The coverage and lengths of them are similarly evaluated. We also randomly order the hypotheses $(k_1,\dots,k_t)$ and evaluate for ten times.

Worst-case method. Similar to the previous worst-case method, we will calibrate an upper bound of conditional shift and compute prediction intervals. Here, the upper bound will be calibrated with the observed data. Specifically, given all sites for hypotheses $\{k_1, \ldots, k_t\}$, we compute individual KL divergences following SI Appendix 2.D.1, and then compute

$$\hat{\mathrm{KL}}_{\mathrm{upp}}^{(t)} := \mathrm{Quantile}\Big(0.99;\ \{\hat{\mathrm{KL}}_{i\to j}^{(k_s)}\}_{i\neq j, s\leq t}\Big). \tag{8}$$

For each future site pair (i, j) for hypothesis k_s, $s > t$, we solve an empirical version of

$$\begin{aligned} \text{Maximize/minimize} \quad & \theta(\bar{Q}) \\ \text{Subject to} \quad & \mathrm{KL}\big(\bar{Q} \,\|\, P_{j,X}^{(k_s)} \times P_{i,Y|X}^{(k_s)}\big) \leq \hat{\mathrm{KL}}_{\mathrm{upp}}^{(t)}, \end{aligned}$$

and use the obtained maximized/minimized objectives as the upper/lower bounds. Note that this time, all quantities are computable in a real generalization task.

3. Additional empirical results

A. Other calibration scenarios. In this part, we present additional calibration scenarios omitted in Section 3 in the main text, where we use certain observed data to calibrate the relative strength of covariate and conditional shifts, and construct prediction intervals in future generalization tasks. We omit the detailed procedures as they follow exactly the same ideas as Appendix 2.D.2, except for the construction of the bounds L and U in [7].

Calibration with other sites. The second scenario is to calibrate the measures with existing sites involving all hypotheses for new sites. We randomly order the sites with $(j_1, \ldots, j_N)$ as a permutation of $(1, \ldots, N)$. Then, at each step $t \in \{1, \ldots, N-1\}$, we assume data from sites $\{j_1, \ldots, j_t\}$ for all the hypotheses are observed, and use the empirical quantiles of $\{\hat{r}_{i\to j}^{(k)}\}_{i,j\in\{j_1,\ldots,j_t\},k=1,\ldots,K}$ as L and U in the construction of prediction intervals [9]. Finally, for each pair of sites $j_1, j_2 \in \{j_{t+1}, \ldots, j_{29}\}$, we consider the task of generalization from fully observed data in site j_1 for hypothesis k to partially observed site j_2 for all hypotheses $j \in \{1, \ldots, 10\}$, using the aforementioned quantiles to construct prediction intervals following [9]. On the other hand, the KL-divergence bound for `WorstCase` is also calibrated with these existing pairs in a way that is similar to [8].

The empirical coverage and PI lengths calibrated with other sites are reported in Figure S1. Again, the `WorstCase` method exhibits overcoverage and very wide intervals, while our method achieves valid coverage while being close to the `Oracle` method.

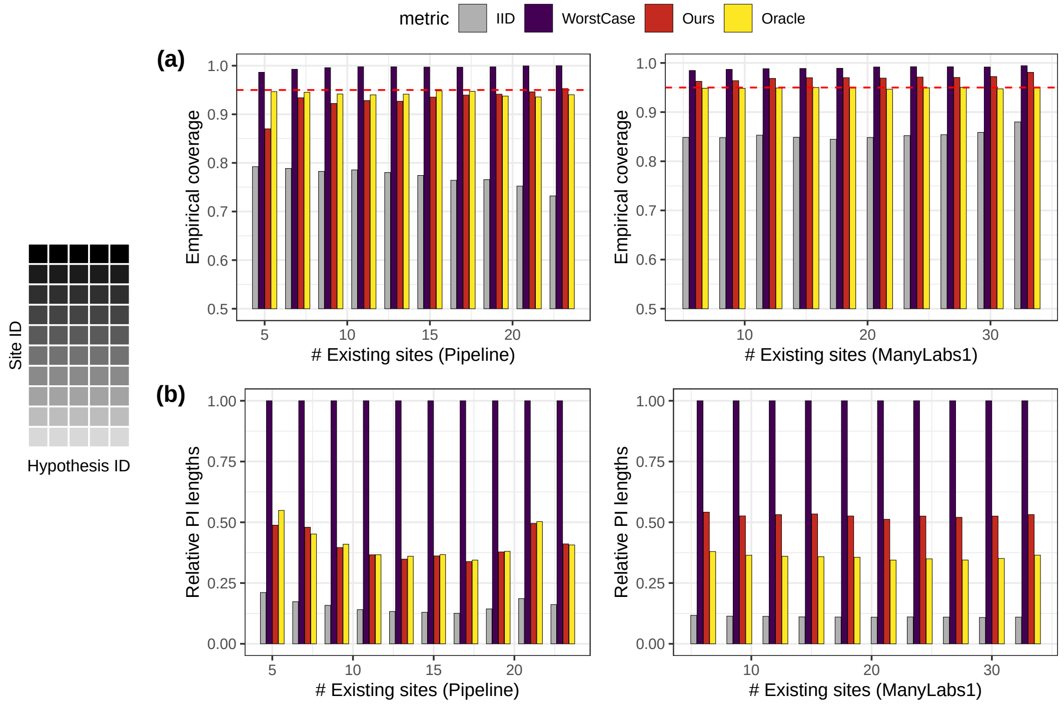

Fig. S1. *Generalization in new studies based on distribution shift measures from other sites.* **Left**: *Illustration of data collection order, where dark color means earlier.* **Row (a)**: *Average coverage of prediction intervals using the Pipeline data (left) and ManyLabs 1 data (right).* **Row (b)**: *Average length of prediction intervals using the Pipeline data (left) and ManyLabs 1 data (right). Details are otherwise as Figure 8.*

Calibration with other sites and other hypotheses. The final scenario is the most challenging, where for a new generalization task, only data from other sites for other hypotheses are available. Specifically, we randomly order the sites by $(j_1, \ldots, j_N)$ and hypotheses by $(k_1, \ldots, k_{10})$. Then, at each step $t \in \{1, \ldots, 9\}$, data for studies $\{k_1, \ldots, k_K\}$ are available in sites $\{j_1, \ldots, j_{3t}\}$, we use the empirical quantiles of $\{\hat{r}^{(k)}_{i\to j}\}_{i,j\in\{j_1,\ldots,j_{3t}\},k\in\{k_1,\ldots,k_K\}}$ as L and U in the construction of prediction intervals [9]. Finally, for each pair of sites $j_1, j_2 \in \{j_{3t+1}, \ldots, j_N\}$, we consider generalization from site j_1 to site j_2 for each hypothesis $k \in \{k_{t+1}, \ldots, k_K\}$, using the aforementioned quantiles to construct prediction intervals following [9]. The KL-divergence bound for `WorstCase` is also calibrated with these existing pairs similar to [8].

The empirical coverage and length of PIs are reported in Figure S2. Similar to the observations in other scenarios, `WorstCase` is much more conservative, while our method achieves valid coverage with prediction interval lengths close to `Oracle`. This scenario is the most challenging among all, since the sites and hypotheses are entirely disjoint between existing data and new generalization tasks.

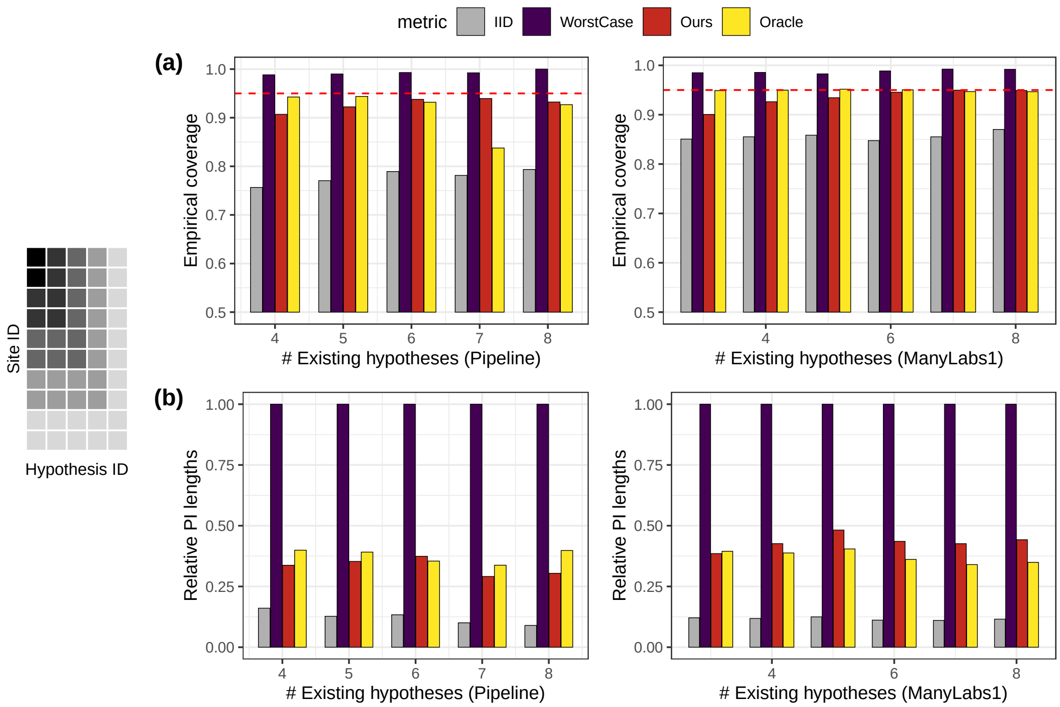

Fig. S2. *Generalization in new studies based on distribution shift measures from other sites and other hypotheses for new sites and new hypotheses.* **Left**: *Illustration of data collection order, where dark color means earlier.* **Row (a)**: *Average coverage of prediction intervals using the Pipeline data (left) and ManyLabs 1 data (right).* **Row (b)**: *Average length of prediction intervals using the Pipeline data (left) and ManyLabs 1 data (right). Details are otherwise as Figure 8.*

B. Relative strengths of distribution shift measures. In this part, we report additional results for the relative strengths of distribution shift measures in both projects, which complement Figure 4 in the main text. In particular, Figures S3 and S4 plots distribution shift measures in each hypothesis of the Pipeline project, computed with entropy balancing and the doubly robust estimator, respectively. Figures S5 and S6 plot those for the ManyLabs1 project.

Consistent with Figure 4, we see that the covariate shift upper bounds the conditional shift most of the time, but the balancing method tends to produce more stable estimates with small-to-moderate sample sizes.

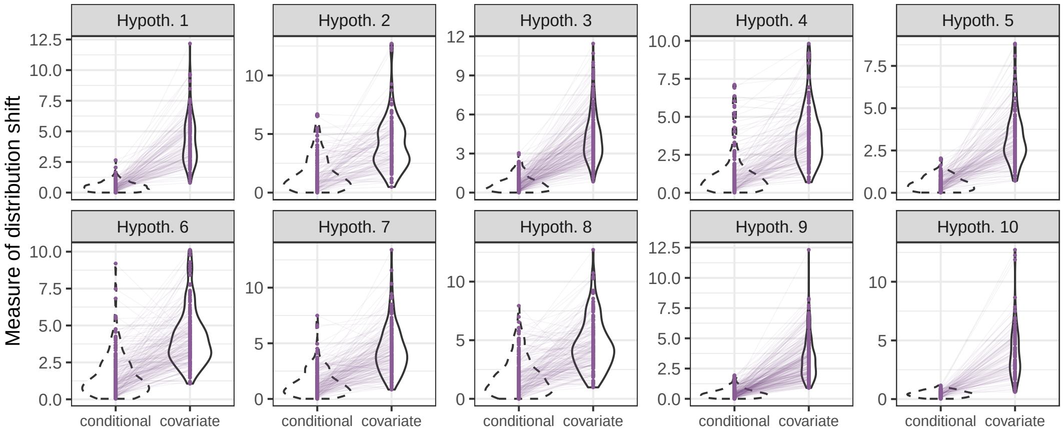

Fig. S3. Distribution shift measures between all site pairs in each hypothesis in the Pipeline project, where the covariate shift adjustment uses entropy balancing.

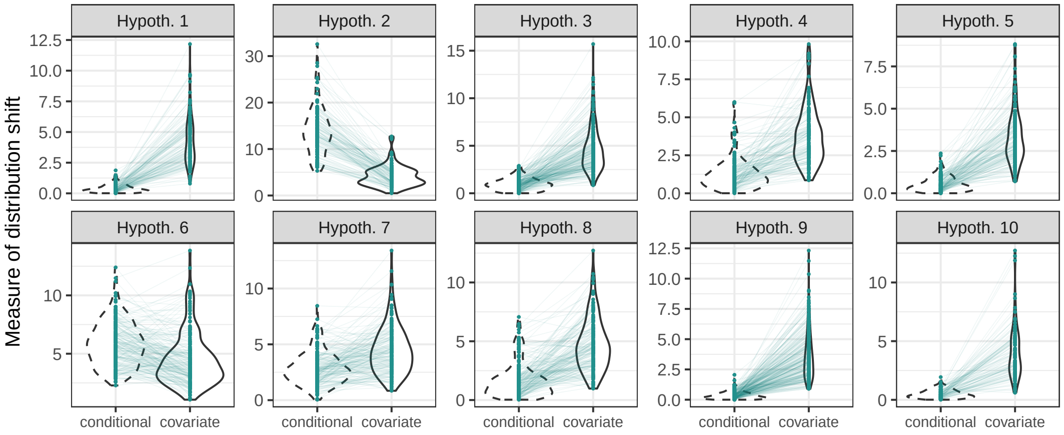

Fig. S4. Distribution shift measures between all site pairs in each hypothesis in the Pipeline project, where the covariate shift adjustment uses the doubly robust estimator.

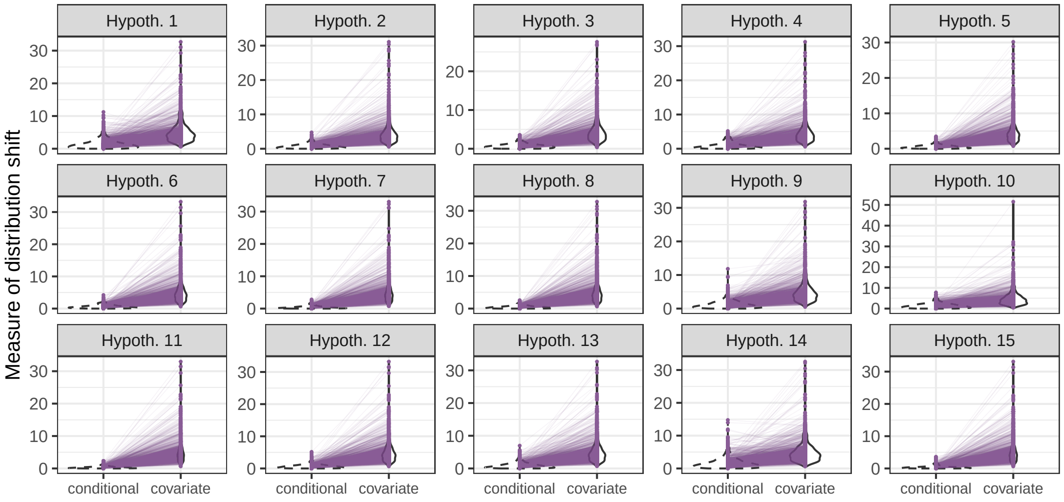

Fig. S5. Distribution shift measures between all site pairs in each hypothesis in the ManyLabs1 project, where the covariate shift adjustment uses entropy balancing.

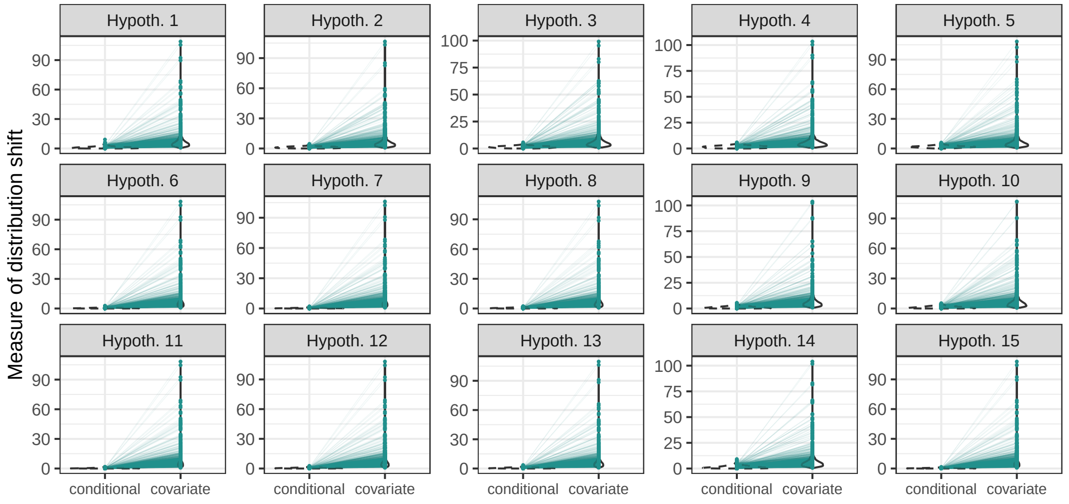

Fig. S6. Distribution shift measures between all site pairs in each hypothesis in the ManyLabs1 project, where the covariate shift adjustment uses the doubly robust estimator.

4. Exploring alternative distribution shift measures

This selection collects our results on exploring alternative distribution shift measures. Here we exclusively focus on entropy-balancing-based estimation for stability and conciseness, while doubly-robust estimation exhibits similar patterns. Also, we only present results for the Pipeline project for conciseness.

We introduce two sets of alternative distribution shift measures. By comparing our measures in the main text with them, we demonstrate the importance of (i) re-scaling by standard deviation to ensure scale invariance, and (ii) stabilizing the covariate shift measure in our definitions in Section 2.A in the main text.

A. Alternative distribution shift measures. Following the notations in Section 2.A in the main text, we consider generalizing from a site with distribution P to a site with distribution Q, and the parameter of interest has an influence function ϕ. Recall that $\phi_P(x) := \mathbb{E}_P[\phi \,|\, X = x]$.

Marginal shift measures. The first set of distribution shift measures follow (14), which are not rescaled by the standard deviation. Namely,

$$\text{absolute of conditional shift} \;=\; \mathbb{E}_Q[\phi(T,Y) - \phi_P(X)],$$
$$\text{absolute covariate shift} \;=\; \mathbb{E}_Q[\phi_P(X)] - \mathbb{E}_P[\phi_P(X)].$$

These quantities describe the contributions of various types of distribution shifts to the discrepancy between effect estimates from two studies (sites) in (14). Their estimation is already included in Appendix 2.C, following which we denote the estimators as $\hat{\Delta}_{Y\,|\,X}$ and $\hat{\Delta}_X$, respectively. Namely,

$$\hat{\Delta}_{Y\,|\,X}^{i\to j,(k)} := \hat{\theta}_j^{(k)} - \hat{\theta}_{i\to j}^{(k)}, \qquad [9]$$
$$\hat{\Delta}_{X}^{i\to j,(k)} := \hat{\theta}_{i\to j}^{(k)} - \hat{\theta}_i^{(k)}, \qquad [10]$$

where $\hat{\theta}_{i\to j}^{(k)}$ is the reweighted estimator using full observations from site i and covariates from site j.

These unscaled measures may lack interpretability in certain cases. For one thing, the magnitude of these quantities depends on how sensitive the function ϕ is to shifts in the probability space: for instance, if $\phi(X)$ is highly heterogeneous, then even small changes in the distribution of X would lead to large values of absolute covariate shift. While this is meaningful for diagnosing how the effect discrepancy relies on the distribution shifts and guiding future data collection efforts as in (14), this might be undesirable when we are interested in *understanding the distribution shift itself.*

We will see later that with marginal shift measures, the conditional shift is usually much larger than the covariate shift measure, which is consistent with the (somewhat pessimistic) findings in (14) and a similar work of (13). This is mainly due to the fact that $\mathrm{sd}(\phi - \phi_P(X))$ is much larger than $\mathrm{sd}(\phi_P(X))$, i.e., the explanatory power of X for the parameter is low. However, this hides the fact that the strength of perturbation to the probability space is indeed the other way.

Relative shift measures. The second set of distribution shift measures follow Section 2.A in the main text, but we adopt the relative conditional shift instead of the stabilized one. Thus, we call them relative shift measures. The estimation of the relative conditional shift is straightforward; following Appendix 2.C, we use

$$\hat{\Delta}_{\mathrm{rel},X}^{i\to j,(k)} = \frac{\hat{\Delta}_X^{i\to j,(k)}}{\hat{s}_{i,X}} = \frac{\hat{\theta}_{i\to j}^{(k)} - \hat{\theta}_i^{(k)}}{\hat{s}_X^{i\to j,(k)}}, \qquad [11]$$

where $\hat{\theta}^{(k)}_{i\to j}$ is the reweighted estimator using entropy balancing or doubly robust estimator, and $\hat{s}_X^{i\to j,(k)}$ is a consistent estimator for $\mathrm{sd}_P(\phi_P(X))$ which can be obtained following the estimation of $\mathrm{sd}_P(\phi - \phi_P(X))$.

The issue with $\hat{\Delta}^{i\to j,(k)}_{\mathrm{rel},X}$ is that the quantity $\hat{s}_X^{i\to j,(k)}$ can be extremely small in some cases when the explanatory power of X for ϕ is low, as typical in the datasets we study here. Thus, even if we also observe a bounded role of relative covariate shift for relative conditional shift, the estimation is so unstable that it is not appropriate to be used in generalization tasks.

Summary. We summarize the three sets of distribution shift measures in Table S7 for the ease of reference. We also include the notations for their ratios to be used in the next two subsections.

Name	Conditional shift measure	Covariate Shift measure	Shift ratio
Stabilized	$\hat{t}_{Y\mid X}$, [5]	$\hat{t}_{Y\mid X}$, [6]	$\hat{r}^{\mathrm{stab}}$
Relative	$\hat{t}_{Y\mid X}$, [5]	$\hat{\Delta}_{\mathrm{rel},X}$, [11]	$\hat{r}^{\mathrm{rel}}$
Marginal	$\hat{\Delta}_{Y\mid X}$, [9]	$\hat{\Delta}_X$, [10]	$\hat{r}^{\mathrm{mgn}}$

Table S7. Summary of notations and estimations of distribution shift measures.

B. The importance of rescaling for the predictive role. In this part, we demonstrate that rescaling is important for revealing the predictive role of covariate shift for the unknown conditional shift.

Figure S7 plots the distribution (violin plots) and pairwise relations (connected segments) of each pair of distribution shift measures in Table S7 across all pairs of sites for Hypothesis 5 in the Pipeline project:

- First, in the left panel, the relationship between the marginal measures $\hat{\Delta}_{Y\mid X}$ and $\hat{\Delta}_X$ in each pair is somewhat arbitrary. This means knowledge of $\hat{\Delta}_X$ does not necessarily allow to control $\hat{\Delta}_{Y\mid X}$.

- The middle panel of Figure S7 shows that the relative measure of covariate shift $\hat{\Delta}_{\mathrm{rel},X}$ bounds the conditional shift measure $\hat{t}_{Y\mid X}$ most of the time. This reveals the importance of normalization with standard deviation for interpretability. Without being scale-invariant, the marginal measures fail to reveal the predictive role since the conditional "sensitivity", quantified by $\mathrm{sd}(\phi - \phi_P(X))$, is larger than $\mathrm{sd}(\phi_P(X))$. However, estimated values of $\hat{\Delta}_{\mathrm{rel},X}$ can be extremely large, since $\mathrm{sd}(\phi_P(X))$ and its estimated value can be tiny when the explanatory power of the covariates is low. This is also not desirable in practice as it will cause instability in downstream tasks such as effect generalization; we will explore this in the next part.

- Finally, the right panel of shows the stabilized measures introduced in the main text. They reveal the predictive role of covariate shift due to scale invariance; in addition, they are more stable than the relative measures, and the bounding role is tighter due to fewer extreme estimated values.

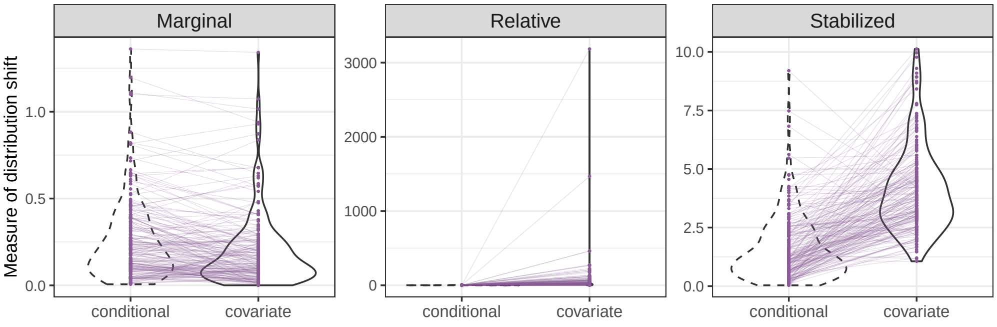

Fig. S7. *(Relative) magnitude of measures of conditional shift (dashed) and covariate shift (solid) across all site pairs in hypothesis 5 of the Pipeline project, analyzed with entropy balancing.* **Left**: *Marginal measures* $\hat{\Delta}_{Y\mid X}$ *and* $\hat{\Delta}_X$. **Middle**: *Relative measures* $\hat{t}_{Y\mid X}$ *and* $\hat{\Delta}_{\mathrm{rel},X}$. **Right**: *Stabilized measures* $\hat{t}_{Y\mid X}$ *and* $\hat{t}_X$.

With a similar goal as panel (c) of Figure 4 in the main text, we explore the stability of the three sets of distribution shift ratios by their within-hypothesis quantiles. If quantiles of the ratios are stable across hypotheses, then the ratio is "pivotal" and generalizable, meaning that external knowledge of the magnitude of distribution shift ratios from other data sources may be useful for the data at hand. From Figure 4, we see that the quantiles of the marginal ratios and ralative ratios are quite variable. In contrast, the within-hypothesis quantiles of the stabilized ratio are more "pivotal"; they are stable across hypotheses and also close to the global quantile. We will see next the implications of such stability for generalization.

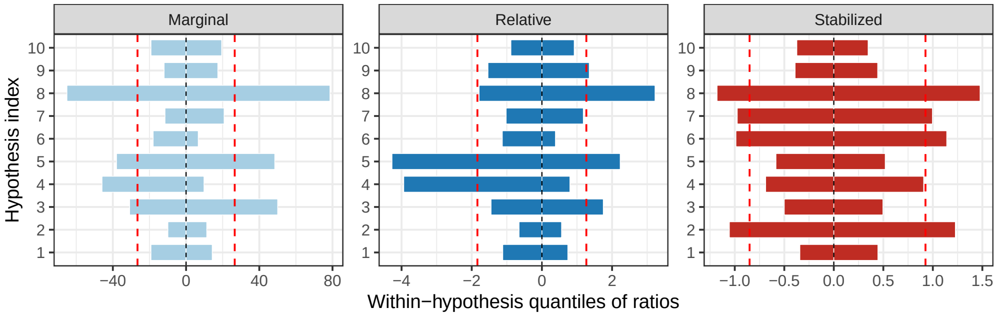

Fig. S8. *Lower and upper within-hypothesis quantiles of ratios which, once known, lead to exact* 95% *empirical coverage of the prediction intervals for the Pipeline dataset. The left ends of the bar plot are the lower quantiles; the right ends are the upper quantiles. The red dashed lines are the global quantiles over all studies. Ideally, the quantiles should be invariant across studies for meaningful empirical calibration.* **Left**: *quantiles of* $\hat{r}^{\mathrm{mgn}}$ *(marginal);* **Middle**: *quantiles of* $\hat{r}^{\mathrm{rel}}$ *(relative);* **Right**: *quantiles of* $\hat{r}^{\mathrm{stab}}$ *(stabilized).*

C. The importance of stability for generalization. We evaluate generalization tasks similar to Section 3 in the main text with the three sets of distribution shift measures. Again, similar to the ideas of [9], we construct prediction intervals for the target site estimator $\hat{\theta}_j^{(k)}$ by calibrating lower and upper bounds for the ratios between each suite of distribution shifts. The detailed estimation procedures follow Appendix 2.D.

Specifically, we aim to find lower and upper bounds for the ratios, such that (approximately)

$$\mathbb{P}\Big(L^{\mathrm{mgn}} \le \hat{r}_{i\to j}^{\mathrm{mgn},(k)} \le U^{\mathrm{mgn}}\Big) \ge 1-\alpha, \quad \hat{r}_{i\to j}^{\mathrm{mgn},(k)} = \hat{\Delta}_{Y|X}^{i\to j,(k)} / \hat{\Delta}_{X}^{i\to j,(k)}, \tag{12}$$
$$\mathbb{P}\Big(L^{\mathrm{rel}} \le \hat{r}_{i\to j}^{\mathrm{rel},(k)} \le U^{\mathrm{rel}}\Big) \ge 1-\alpha, \quad \hat{r}_{i\to j}^{\mathrm{rel},(k)} = \hat{t}_{Y|X}^{i\to j,(k)} / \hat{\Delta}_{\mathrm{rel},X}^{i\to j,(k)},$$

and the bounds for the ratio between stabilized measures in the main text follow the idea of [A].

Inverting the events in [12] and by the definition of the measures, we set the prediction intervals

$$\hat{C}_{i\to j}^{\mathrm{mgn},(k)} := \Big[\; \hat{\theta}_{i\to j}^{(k)} + \hat{\Delta}_{X}^{i\to j,(k)} \cdot L^{\mathrm{mgn}},\; \hat{\theta}_{i\to j}^{(k)} + \hat{\Delta}_{X}^{i\to j,(k)} \cdot U^{\mathrm{mgn}} \;\Big]$$
$$\hat{C}_{i\to j}^{\mathrm{rel},(k)} := \Big[\; \hat{\theta}_{i\to j}^{(k)} + \hat{\Delta}_{\mathrm{rel},X}^{i\to j,(k)} \cdot \hat{s}_{Y|X}^{i\to j,(k)} \cdot L^{\mathrm{rel}},\; \hat{\theta}_{i\to j}^{(k)} + \hat{\Delta}_{\mathrm{rel},X}^{i\to j,(k)} \cdot \hat{s}_{Y|X}^{i\to j,(k)} \cdot U^{\mathrm{rel}} \;\Big],$$

and recall that $\hat{C}_{i\to j}^{(k)}$ is the prediction interval [9] based on our shift measures in the main text. We then evaluate the empirical coverage and average length of these prediction intervals.

Oracle calibration. For reference, we evaluate the `Oracle` method in the main text for the three sets of shift measures, in order to show their performance in the most ideal case where the distribution of their ratios is perfectly known. Here, the L and U values in [12] are the empirical quantiles of the shift ratios between all site pairs within each hypothesis. The empirical coverage and average length of prediction intervals within each hypothesis are in Figure S9. All three sets of measures lead to perfect 0.95 coverage as expected. However, the prediction intervals by the stabilized measures several folds shorter (the y-axis is log-scaled for easier visualization), showing the importance of estimation stability.

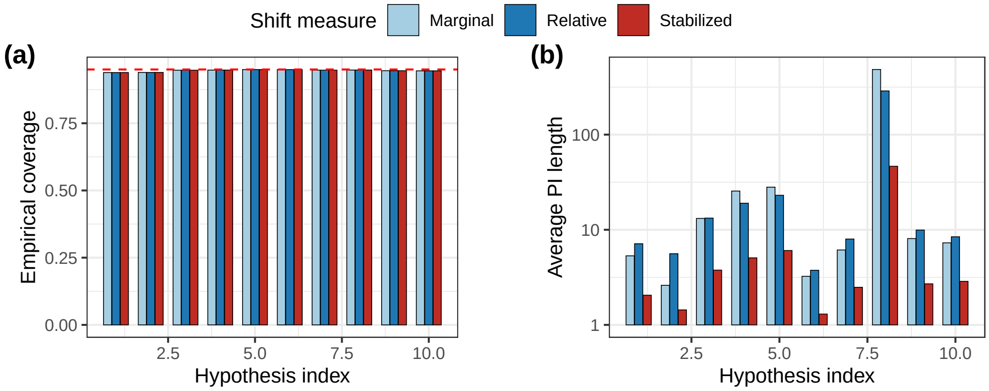

Fig. S9. Left: *Empirical coverage of oracle calibrated prediction intervals at nominal level* $1-\alpha = 0.95$*. The coverage is ensured to be* 95% *since full observations are used.* **Right**: *Average length of prediction intervals for in-study calibrated prediction intervals at nominal level* $1-\alpha = 0.95$ *based on three measures. The* y*-axis on the right is log-scaled for visualization.*

Constant calibration. Similar to Section 3 in the main text, here we simply take all three lower quantiles to be -1, and all three upper quantiles to be 1, with the belief that the covariate shift measure upper bounds the conditional shift measure. The hypothesis-wise coverage and average length of constant-calibrated prediction intervals are in Figure S10. It is not surprising that assuming $|\hat{\Delta}_{Y|X}| \leq |\hat{\Delta}_X|$ leads to poor coverage (marginal). Assuming that the conditional shift measure is bounded by the covariate shift measure leads to satisfying coverage for both the relative and stabilized measure. However, the stabilized measures lead to much shorter prediction intervals and slightly better coverage again due to stability.

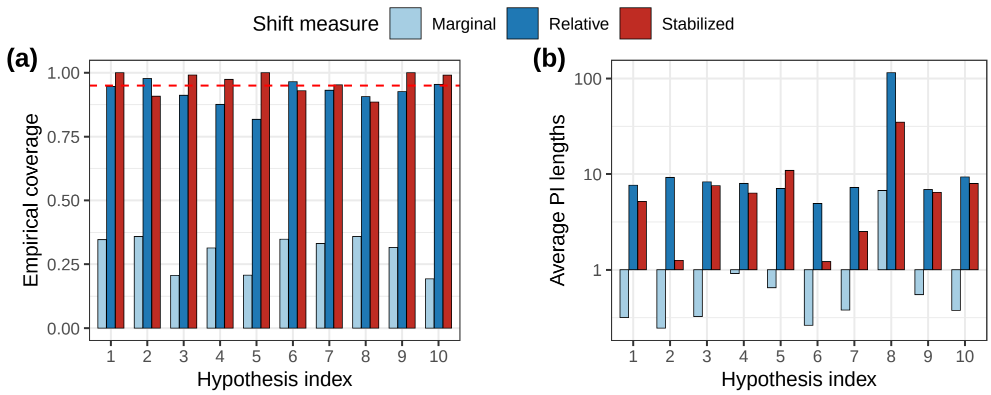

Fig. S10. **Left**: *Empirical coverage of constant calibrated prediction intervals at nominal level* $1-\alpha=0.95$. **Right**: *Average length of prediction intervals for constant calibrated prediction intervals at nominal level* $1-\alpha=0.95$ *based on three measures. The* y*-axis on the right is log-scaled for visualization.*

Data-adaptive calibration. Finally, we consider the data-adaptive calibration scenario where full observations from other sites/hypotheses are available, which are used to compute the quantiles for a new generalization task. The specific methods are the same as Section 3 in the main text and SI Appendix 3.A, with detailed procedures following Appendix 2.D.

The first scenario is the same as Section 3 in the main text, where data for some other hypotheses in all sites are available, and the new generalization task involves new hypotheses. Figure S11 illustrates the order of data collection, as well as the coverage and length of prediction intervals, averaged over 10 random draws of hypothesis ordering. We see that all three measures lead to satisfactory coverage, meaning that *the distribution shift measures tend to be "generalizable" across hypotheses/estimators/outcomes*. Yet, the stabilized measures still yield the shortest prediction intervals.

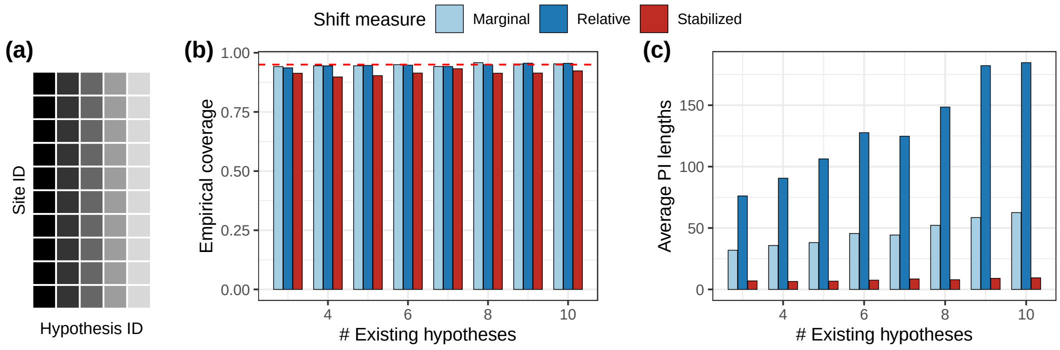

Fig. S11. *Generalization based on distribution shift measures calibrated with data for other hypotheses in the same sites.* **Left**: *Illustration of data collection order, where dark color means earlier.* **Middle**: *Average coverage (bars) of prediction intervals over 10 random draws of study ordering. The red dashed line is the nominal level* 0.95. **Right**: *Average length of prediction intervals based on three sets of shift measures.*

The second scenario is to calibrate the measures with existing sites involving all hypotheses for new sites, same as "calibration with other sites" in Appendix 3.A. Figure S12 presents the corresponding results. We see that all measures lead to satisfactory coverage, while the stabilized measures lead to much shorter prediction intervals.

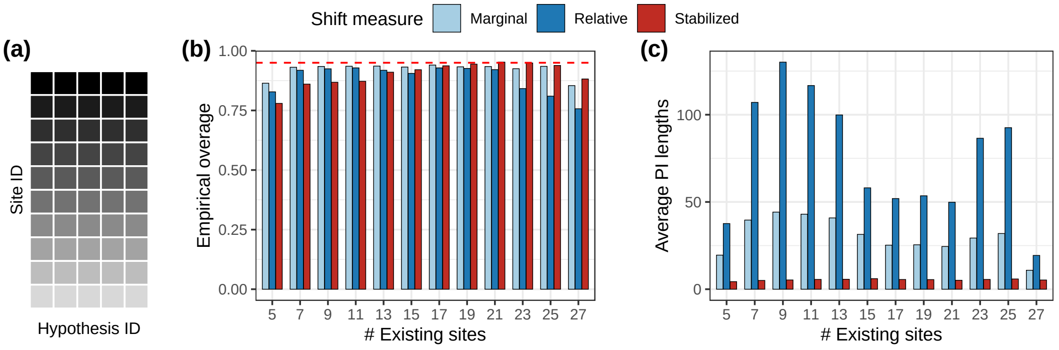

Fig. S12. *Data collection order, average coverage and length of prediction intervals for generalization in new sites based on data from the same studies in other sites. Details are otherwise the same as in Figure S11.*

Finally, we use data from other sites for other hypotheses to calibrate the upper and lower bounds for the distribution shift measures, which is the same as "Calibration with other sites and other hypothesis" in Appendix 3.A. Figure S13 presents the results for the third scenario. Due to the limited samples, we observe slight undercoverage when only two hypotheses and sites are available. Similar to other scenarios, the stabilized measure leads to much shorter prediction intervals than the other two.

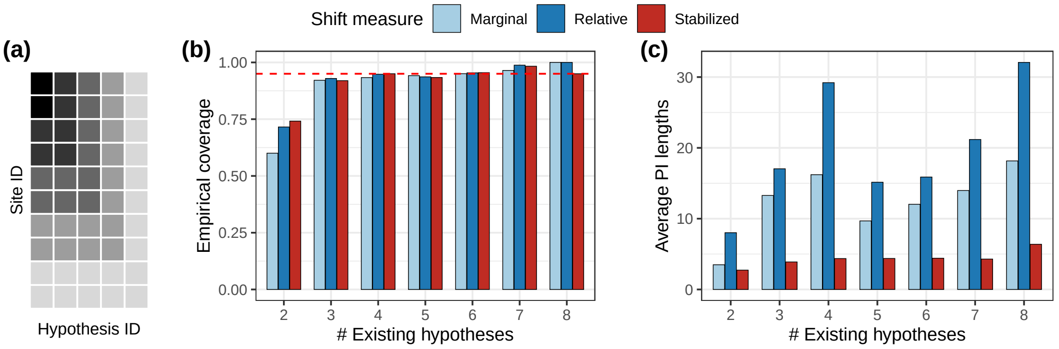

Fig. S13. *Data collection order, average coverage and length of prediction intervals for generalization between new sites in new studies based on data for other studies from other sites. Details are otherwise as in Figure S11.*

5. Deferred discussion, technical details, and proofs

A. Connection to a hierarchical random-effect-type model. In the main text, we explain the predictive role of covariate shift for the unknown conditional shift. While our empirical findings hold across various contexts, our theoretical model might feel a bit abstract at the first sight. Here we note that our random distribution shift model is just one of the possible models that may explain the empirical phenomenon, and many messages can be contextualized with commonly used models.

In the following, we connect our model to a random effects–type model, which is widely used in meta-analyses and replication studies. In this familiar framework, a similar phenomenon (that shift in X predicts shift in Y) can arise when the perturbations to the covariates X and the unobserved effect modifiers U are of comparable magnitude.

Following ideas of the random effect model, we consider a two-stage hierarchical model (this is arguably more complex than the common random effects model describing the *effect sizes* only, but we specify the distributions for the purpose of explicitly describing distribution shift). Under the distribution $P^{(k)}$ of site k, we assume the observed covariates X and unobserved modifiers U are independent with $X \sim N(\mu_k^X, \mathbf{I})$, $U \sim N(\mu_k^U, \mathbf{I})$ where $\mu_k^X, \mu_k^U \in \mathbb{R}^d$. For simplicity, we consider the case where we are interested in the mean of Y (instead of the causal effect) and posit the linear model $Y = \alpha^\top X + \beta^\top U$. Then,

$$t_X := \text{stabilized covariate shift} = \sqrt{\frac{1}{d}\sum_{j=1}^{d}\frac{(\mathbb{E}_Q[X_j] - \mathbb{E}_P[X_j])^2}{\mathrm{Var}_P(X_j)}} = \frac{\|\mu_k^X - \mu_\ell^X\|_2}{\sqrt{d}},$$

$$t_{Y|X} := \text{relative conditional shift} = \frac{E_Q[Y - E_P[Y|X]]}{\mathrm{sd}(Y - E_P[Y|X])} = \frac{\beta^\top(\mu_k^U - \mu_\ell^U)}{\|\beta\|_2}.$$

The distribution shift between sites (k, ℓ) is captured by the differences in the parameters (μ_k^X, μ_k^U) and (μ_ℓ^X, μ_ℓ^U). Note that the changes in $P(x)$ is driven by $\{\mu_k^X\}_{k=1}^K$, whereas the changes in $P(y \mid x)$ is driven by $\{\mu_k^U\}_{k=1}^K$ (and β). As in random-effect-type models, we assume (μ_k^X, μ_k^U) is drawn from a certain joint distribution. In the following, we explore the impact of the joint distribution of (μ_k^X, μ_k^U).

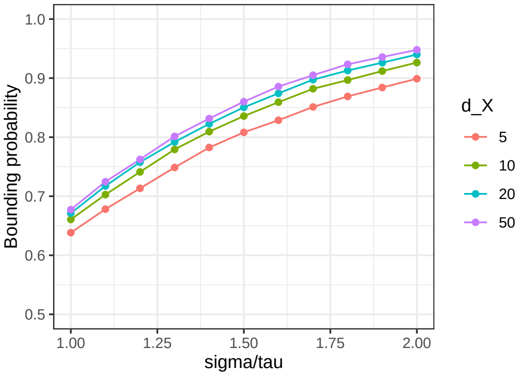

Fig. S14. Probability of $t_X \geq t_{Y|X}$ for various values of $(d, \sigma/\tau)$.

To fix ideas, we for now assume $\mu_k^X \sim N(0, \sigma^2 \mathbf{I})$ and $\mu_k^U \sim N(0, \tau^2 \mathbf{I})$ are independent. Then, marginally over the distributional uncertainty, we have

$$t_X \overset{d}{=} \sqrt{2}\sigma\sqrt{\chi_d^2/d}, \quad t_{Y|X} \overset{d}{=} \sqrt{2}\tau Z',$$

where χ_d^2 and $Z' \sim N(0,1)$ are independent standard random variables. Here, the concentration of χ_d^2/d can be characterized by $\sqrt{\chi_d^2/d} = 1 + O_P(1/\sqrt{d})$, thus when d is relatively large, we would have $t_X \approx \sqrt{2}\sigma$, which upper bounds $|t_{Y|X}|$ if $\sigma \geq \tau$ with high probability, leading to a similar predictive role (indeed, t_X implicitly estimates the strength σ). In addition, when the parameter of interest is the treatment effect where the distribution T is invariant, we would have $|t_{Y|X}| \sim \sqrt{2}\tau'|Z|$ for $\tau' < \tau$, leading to an even higher probability of $t_X \geq |t_{Y|X}|$. In Figure S14, we plot the probability of $t_X \geq |t_{Y|X}|$ under different values of d and σ/τ. We see that with $d = 5$ and $\sigma = \tau$, we already have $\mathbb{P}(t_X \geq |t_{Y|X}|) \geq 0.6$, and it gets much higher with moderately large d (i.e., there are d independent perturbations to the X space) and moderately large σ/τ (i.e., the distribution shift in covariates is no smaller than that in hidden effect modifiers). If (μ_k^X, μ_k^U) are correlated, the description of $(t_X, t_{Y|X})$ would need many more parameters, but the same overall pattern applies.

To summarize, in this commonly used random-effects-type model, the covariate shift measure could upper bound the conditional shift measure if the distribution perturbation strength obeys $\sigma \geq \tau$. Essentially, this means that the distribution uncertainty exhibits no preference in perturbing X or U (symmetry, as similar to our random shift model).

Indeed, our model can be viewed as a nonparametric version of the above hierarchical random effects model, and we sketch some high-level ideas on their connections below:

- The many random and unintended factors perturbing the probability space of (X, U) in our model conceptually resemble random sampling of μ_k^X and μ_k^U in the hierarchical model.

- In both models, the standardized covariate shift t_X serves as an estimate for the distribution shift strength. Specifically, in both models, we have $t_X \sim \nu\sqrt{\chi_d^2/d}$ for some $\nu \in \mathbb{R}^+$ and some $d \in \mathbb{N}^+$. In our model, ν is the strength of perturbation depending on the $\{W_i\}$; here, $\nu = \sqrt{2}\sigma$ is the parameter that controls the variance of μ_k^X, which also reflects the strength of covariate shifts.

- In both models, the relative conditional shift $|t_{Y|X}| \approx \nu' \cdot |Z|$ for some $\nu' \in \mathbb{R}^+$ and $Z \sim N(0,1)$ up to higher-order errors. Once we assume the perturbations to X and U are comparable, we have $\nu' \leq \nu$ and yield the fact that $|t_{Y|X}|$ can be upper bounded by t_X with high probability (explaining our empirical observations).

In addition, using a nonparametric model has several benefits:

- It requires less model specification efforts while reaching similar intuitive interpretations.

- Unlike meta analysis which typically needs a moderate number of studies, using the predictive role of covariate shift enables estimation and generalization with only *a pair of data*, making it more suitable for generalization tasks.

B. Proof of distributional CLT.

Proof. Let $n_Q(M)$ and $n_P(M)$ be sequences of natural numbers such that $n_Q(M)/M$ and $n_P(M)/M$ converge to positive real numbers. In the following, for simplicity, we will suppress the dependence of n_Q and n_P on M. We will first show the result for bounded, one-dimensional ϕ with $\mathbb{E}_P[\phi] = 0$. Define $\sigma_M^2 = \frac{1}{M}\sum_{m=1}^M \mathbb{E}_P[\phi | C_m^{(M)}]^2 - \mathbb{E}_P[\phi]^2$. As $M \to \infty$, by assumption we have $\sigma_M^2 \to \mathrm{Var}_P(\mathbb{E}_P[\phi|X,U]) =: \sigma^2$. If $\mathrm{Var}_P(\mathbb{E}_P[\phi|X,U]) = 0$, then $\mathbb{E}_P[\phi] = \mathbb{E}_Q[\phi]$, thus only uncertainty due to i.i.d.

sampling remains and the statement of the theorem is trivial. Thus, in the following we will assume $\mathrm{Var}_P(\mathbb{E}_P[\phi|X,U]) > 0$. We can use $\mathbb{E}_P[\phi] = \frac{1}{M}\sum_{m=1}^M \mathbb{E}_P[\phi|C_m^{(M)}]$ to obtain

$$\begin{aligned}
&\frac{\sqrt{M}(\mathbb{E}_Q[\phi]-\mathbb{E}_P[\phi])}{\sigma_M \mathrm{sd}(W)/E[W]} \\
&= \frac{\frac{M^{-1/2}\sum_{m=1}^M (W_m - 1/M\sum_{m'} W_{m'})\mathbb{E}_P[\phi|C_m^{(M)}]}{1/M\sum_m W_m}}{\sigma_M \mathrm{sd}(W)/E[W]} \\
&= \frac{M^{-1/2}\frac{\sum_{m=1}^M (W_m - 1/M\sum_{m'} W_{m'})\mathbb{E}_P[\phi|C_m^{(M)}]}{E[W]}}{\sigma_M \mathrm{sd}(W)/E[W]} + o_P(1/\sqrt{M}) \\
&= \frac{M^{-1/2}\sum_{m=1}^M (W_m - \mathbb{E}[W])\mathbb{E}_P[\phi|C_m^{(M)}]}{\sigma_M \mathrm{sd}(W)} + o_P(1/\sqrt{M}).
\end{aligned}$$

We will now check Lindeberg's condition with $v_M = \sum_{m=1}^M \mathbb{E}_P[\phi|C_m^{(M)}]^2 - \mathbb{E}_P[\phi]^2$. By assumption $v_M/M \to \sigma^2 > 0$. Furthermore, by assumption $|\phi|_\infty \le B$ for some constant $B > 0$. Let $\epsilon > 0$. Then

$$\begin{aligned}
&\limsup_{M\to\infty} \frac{1}{v_M}\mathbb{E}[\sum_{m=1}^M (W_m - \mathbb{E}[W])^2(\mathbb{E}_P[\phi|C_m^{(M)}] - \mathbb{E}_P[\phi])^2 1_{|W_m - \mathbb{E}[W]||\mathbb{E}_P[\phi|C_m^{(M)}] - \mathbb{E}_P[\phi]| \ge \epsilon\sqrt{v_M}}] \\
&\le \limsup_{M\to\infty} \frac{1}{M\sigma^2}\mathbb{E}[\sum_{m=1}^M (W_m - \mathbb{E}[W])^2 4B^2 1_{|W_m - \mathbb{E}[W]| \ge \sqrt{M}\sigma\epsilon/(4B)}] \\
&\le \limsup_{M\to\infty} \frac{4B^2}{\sigma^2}\mathbb{E}[(W - \mathbb{E}[W])^2 1_{|W - \mathbb{E}[W]| \ge \sqrt{M}\sigma\epsilon/(4B)}]
\end{aligned}$$

By dominated convergence, this term is zero. Thus, by Lindeberg's CLT,

$$\frac{\sqrt{M}(\mathbb{E}_Q[\phi]-\mathbb{E}_P[\phi])}{\sigma_M \mathrm{sd}(W)/E[W]} \xrightarrow{d} \mathcal{N}(0,1).$$

By Slutsky, we get the distributional CLT

$$\frac{\sqrt{M}(\mathbb{E}_P[\phi]-\mathbb{E}_Q[\phi])}{\mathrm{sd}_P(\mathbb{E}_P[\phi|X,U])\mathrm{sd}(W)/E[W]} \xrightarrow{d} \mathcal{N}(0,1).$$

We will now combine this result with uncertainty due to i.i.d. sampling. Recall that we consider the case where sampling uncertainty and distributional uncertainty are of the same order, i.e. n_Q/M and n_P/M converge to some positive constants.

$$\begin{aligned}
&\hat{\mathbb{E}}_P[\phi(T,D)] - \hat{\mathbb{E}}_Q[\phi(T,D)] \\
&= \underbrace{\hat{\mathbb{E}}_P[\phi(T,D)] - \mathbb{E}_P[\phi(T,D)]}_{\text{use standard CLT for i.i.d. data}} - \underbrace{(\hat{\mathbb{E}}_Q[\phi(T,D)] - \mathbb{E}_Q[\phi(T,D)])}_{\text{use Berry-Esseen}} + \underbrace{\mathbb{E}_P[\phi(T,D)] - \mathbb{E}_Q[\phi(T,D)]}_{\text{use distributional CLT}}
\end{aligned}$$

We can apply a standard CLT to the first term, since P is fixed and the sample mean $\hat{\mathbb{E}}_P[\phi(T,D)]$ is independent of the remaining terms. For the remaining terms, one complication is that the distribution Q is not fixed, but shifts randomly.

Recall that for now we focus on bounded ϕ, which implies bounded third moments. We will now use Berry–Esseen. For any $x \in \mathbb{R}$, conditionally on the random shift $(W_m)_{m=1,\ldots,M}$,

$$\sup_x \left| P\left(\frac{\sqrt{n_Q}}{\mathrm{sd}_Q(\phi)}(\hat{\mathbb{E}}_Q[\phi(T,D)] - \mathbb{E}_Q[\phi(T,D)]) \le x \middle| (W_m)_{m=1,\ldots,M} \right) - \Phi(x) \right| \le \frac{7.59\mathbb{E}_Q[|\phi|^3]}{\mathrm{sd}_Q(\phi)^3\sqrt{n_Q}}$$

By assumption ϕ is bounded, and by the distributional CLT above $\mathrm{sd}_Q(\phi) \xrightarrow{P} \mathrm{sd}_P(\phi) > 0$ for $M \to \infty$. Thus, conditionally on the random shift $(W_m)_{m=1,\ldots,M}$,

$$\sup_x \left| P\left(\frac{\sqrt{n_Q}}{\mathrm{sd}_P(\phi)}(\hat{\mathbb{E}}_Q[\phi(T,D)] - \mathbb{E}_Q[\phi(T,D)]) \le x \middle| (W_m)_{m=1,\ldots,M} \right) - \Phi(x) \right| \to 0$$

Define

$$Z := \mathbb{E}_P[\phi(T,D)] - \mathbb{E}_Q[\phi(T,D)],$$

$$Z' := \hat{\mathbb{E}}_Q[\phi(T,D)] - \mathbb{E}_Q[\phi(T,D)].$$

Let $\delta_M^2 = \frac{1}{M}\frac{\mathrm{Var}(W)}{E[W]^2}$. By assumption, $n_Q/M \to \rho$ for some constant $\rho > 0$. Thus $n_Q\delta_K^2 \to \rho \mathrm{Var}(W)/E[W]^2$. As $M \to \infty$, for any $x \in \mathbb{R}$

$$\begin{aligned}
&P\left(\left(\frac{\mathrm{Var}_P(\phi)}{n_Q} + \sigma^2\delta_M^2\right)^{-1/2}(Z + Z') \geq x\right) \\
&= E\left[P\left(\frac{\sqrt{n_Q}}{\mathrm{sd}_P(\phi)}Z' \geq \frac{\sqrt{n_Q}}{\mathrm{sd}_P(\phi)}\left(\frac{\mathrm{Var}_P(\phi)}{n_Q} + \sigma^2\delta_M^2\right)^{1/2} x - \frac{\sqrt{n_Q}}{\mathrm{sd}_P(\phi)}Z \middle| (W_m)_{m=1,\ldots,M}\right)\right] \\
&\overset{\text{Berry-Esseen}}{=} E\left[1 - \Phi\left(\left(1 + \rho\frac{\sigma^2\mathrm{Var}(W)}{\mathrm{Var}_P(\phi)E[W]^2}\right)^{1/2} x - \frac{\sqrt{n_Q}}{\mathrm{sd}_P(\phi)}Z\right)\right] + o(1)
\end{aligned}$$

In the third line, we used that $\mathrm{sd}_Q(\phi) \overset{P}{\to} \mathrm{sd}_P(\phi)$. Here, Φ is the cdf of a standard Gaussian random variable. Using weak convergence of $\sqrt{M}Z \overset{d}{\to} \mathcal{N}(0, \sigma^2\mathrm{Var}(W)/E[W]^2)$, and using that Φ is a continuous bounded function we get

$$\begin{aligned}
&E\left[1 - \Phi\left(\left(1 + \rho\frac{\sigma^2\mathrm{Var}(W)}{\mathrm{Var}_P(\phi)E[W]^2}\right)^{1/2} x - \frac{\sqrt{n_Q}}{\mathrm{sd}_P(\phi)}Z\right)\right] \\
\to &E\left[1 - \Phi\left(\left(1 + \rho\frac{\sigma^2\mathrm{Var}(W)}{\mathrm{Var}_P(\phi)E[W]^2}\right)^{1/2} x - \sqrt{\rho}\cdot\frac{\sigma\mathrm{sd}(W)}{\mathrm{sd}_P(\phi)E[W]}G\right)\right],
\end{aligned}$$

where G is a standard Gaussian random variable. With constant $L = \sqrt{\rho}\sigma\mathrm{sd}(W)/(\mathrm{sd}_P(\phi)E[W])$ we can re-write this as

$$E[1 - \Phi(\sqrt{1+L^2}x - LG)] = P(G' \geq \sqrt{1+L^2}x - LG) = P(\frac{G' + LG}{\sqrt{1+L^2}} \geq x) = 1 - \Phi(x),$$

where G' is a standard Gaussian random variable, independent of G. To summarize,

$$P\left(\left(\frac{\mathrm{Var}_P(\phi)}{n_Q} + \sigma^2\delta_M^2\right)^{-1/2}(Z + Z') \geq x\right) \to 1 - \Phi(x).$$

Since the data from P is independent of the perturbation and the data from Q,

$$\left(\mathrm{Var}_P(\phi)\left(\frac{1}{n_P} + \frac{1}{n_Q}\right) + \sigma^2\delta_M^2\right)^{-1/2}\left(\hat{\mathbb{E}}_P[\phi(T,D)] - \hat{\mathbb{E}}_Q[\phi(T,D)]\right) \overset{d}{\to} \mathcal{N}(0,1). \quad [13]$$

We will now extend this result from bounded functions ϕ to square-integrable functions $\phi \in L^2(P)$. Define the bounded function

$$\phi_b = \phi 1_{|\phi|\leq b} + 1_{|\phi|\geq b}\mathbb{E}_P\left[\phi \middle| |\phi| \geq b\right].$$

We have $\mathbb{E}_P[\phi] = \mathbb{E}_P[\phi_b]$. Applying Chebychev conditionally on the random perturbation,

$$P\left(|\hat{\mathbb{E}}_Q[\phi_b - \phi] - \hat{\mathbb{E}}_Q[\phi_b - \phi]| \geq \epsilon \middle| (W_m)_{m=1,\ldots,M}\right) \leq \frac{\mathrm{Var}_Q(\phi_b - \phi)}{\epsilon^2}\left(\frac{1}{n_P} + \frac{1}{n_Q}\right)$$

Take expectations over the random perturbation $(W_m)_{m=1,\ldots,M}$ we obtain that for all $\epsilon > 0$,

$$P(|\hat{\mathbb{E}}_Q[\phi_b - \phi] - \mathbb{E}_Q[\phi_b - \phi]| \geq \epsilon) \leq \frac{\mathbb{E}_P[(\phi - \phi_b)^2]}{\epsilon^2}\left(\frac{1}{n_P} + \frac{1}{n_Q}\right)$$

Similarly, since $W_m \geq w_0$,

$$|\mathbb{E}_Q[\phi - \phi_b]| = |\sum_{m=1}^{M}\frac{W_m}{\sum_{m'}W_{m'}}\mathbb{E}_P[\phi - \phi_b|C_m]| \leq \frac{1}{Mw_0}|\sum_{m=1}^{M}W_m\mathbb{E}_P[\phi - \phi_b|C_m]|.$$

Applying Chebychev and using that $\mathbb{E}_P[\phi - \phi_b] = 0$, we get

$$P(|\mathbb{E}_Q[\phi - \phi_b]| \geq \epsilon) \leq \frac{\sum_{m=1}^{M}(\mathbb{E}_P[\phi - \phi_b|C_m])^2}{w_0^2\epsilon^2M^2} \leq \frac{\mathrm{Var}_P(\phi - \phi_b)}{w_0^2\epsilon^2M}.$$

In the last equation, we used Jensen's inequality. Combining the two applications of Chebychev,

$$P(|\hat{\mathbb{E}}_Q[\phi - \phi_b]| \geq \epsilon) \leq P(|\mathbb{E}_Q[\phi - \phi_b]| \geq \epsilon/2) + P(|\hat{\mathbb{E}}_Q[\phi - \phi_b] - \mathbb{E}_Q[\phi - \phi_b]| \geq \epsilon/2)$$
$$\leq \frac{4}{\epsilon^2}\left(\frac{1}{n_Q} + \frac{1}{n_P} + \frac{1}{w_0^2 M}\right) \mathrm{Var}_P(\phi - \phi_b)$$

Since by assumption $n_P(M) \sim n_Q(M) \sim M$, for any $\epsilon' > 0$, $\epsilon > 0$, we can choose a bounded function ϕ_b such that $P(\sqrt{M}|\hat{E}_Q[\phi - \phi_b] - E_P[\phi - \phi_b]| \geq \epsilon) \leq \epsilon'$ for $M \to \infty$. Let

$$\sigma_{b,M}^2 = \left(\mathrm{Var}_P(\phi_b)\left(\frac{1}{n_Q} + \frac{1}{n_P}\right) + \mathrm{Var}_P(\mathbb{E}_P[\phi_b|X,U])\delta_M^2\right).$$

Then, for any $\epsilon' > 0$ there exists a $\epsilon > 0$ such that as $M \to \infty$, we have

$$P\left(\sigma_{b,M}^{-1}|\hat{E}_Q[\phi - \phi_b] - E_P[\phi - \phi_b]| \geq \epsilon\right) \leq \epsilon'. \tag{14}$$

For any $\delta > 0$, for $b > 0$ large enough

$$\mathrm{Var}_P(\phi)\left(\frac{1}{n_P} + \frac{1}{n_Q}\right) + \mathrm{Var}_P(\mathbb{E}_P[\phi|X,U])\delta_M^2 \leq (1+\delta)^2 \sigma_{b,M}^2.$$

Then, for $M \to \infty$,

$$\limsup_{M\to\infty} P\left(\left(\mathrm{Var}_P(\phi)\left(\frac{1}{n_P} + \frac{1}{n_Q}\right) + \mathrm{Var}_P(\mathbb{E}_P[\phi|X,U])\delta_M^2\right)^{-1/2}\left(\hat{\mathbb{E}}_P[\phi] - \hat{\mathbb{E}}_Q[\phi]\right) \leq x\right)$$
$$\leq \limsup_{M\to\infty} P\left(\sigma_{b,M}^{-1}\left(\hat{\mathbb{E}}_P[\phi] - \hat{\mathbb{E}}_Q[\phi]\right) \leq \max(x(1+\delta), x)\right)$$
$$\leq \limsup_{M\to\infty} P\left(\sigma_{b,M}^{-1}\left(\hat{\mathbb{E}}_P[\phi_b] - \hat{\mathbb{E}}_Q[\phi_b]\right) \leq \max(x(1+\delta), x) + \epsilon\right)$$
$$+ \limsup_{M\to\infty} P\left(\sigma_{b,M}^{-1}\left|\hat{\mathbb{E}}_P[\phi - \phi_b] - \hat{\mathbb{E}}_Q[\phi - \phi_b]\right| \geq \epsilon\right)$$
$$\leq \Phi(\max(x(1+\delta), x) + \epsilon) + \epsilon'.$$

In the last line, we used equation [13] and equation [14]. Since $\delta > 0$, $\epsilon' > 0$ and $\epsilon > 0$ can be chosen arbitrary small,

$$\limsup_{M\to\infty} P\left(\left(\mathrm{Var}_P(\phi)\left(\frac{1}{n_P} + \frac{1}{n_Q}\right) + \mathrm{Var}_P(\mathbb{E}_P[\phi|X,U])\delta_M^2\right)^{-1/2}\left(\hat{\mathbb{E}}_P[\phi] - \hat{\mathbb{E}}_Q[\phi]\right) \leq x\right) \leq \Phi(x).$$

With an analogous argument,

$$\liminf_{M\to\infty} P\left(\left(\mathrm{Var}_P(\phi)\left(\frac{1}{n_P} + \frac{1}{n_Q}\right) + \mathrm{Var}_P(\mathbb{E}_P[\phi|X,U])\delta_M^2\right)^{-1/2}\left(\hat{\mathbb{E}}_P[\phi] - \hat{\mathbb{E}}_Q[\phi]\right) \leq x\right) \geq \Phi(x).$$

Thus, as $M \to \infty$,

$$\left(\mathrm{Var}_P(\phi)\left(\frac{1}{n_P} + \frac{1}{n_Q}\right) + \mathrm{Var}_P(\mathbb{E}_P[\phi|X,U])\delta_M^2\right)^{-1/2}\left(\hat{\mathbb{E}}_P[\phi] - \hat{\mathbb{E}}_Q[\phi]\right) \xrightarrow{d} \mathcal{N}(0,1).$$

This completes the proof for one-dimensional ϕ. The result for a vector of functions ϕ follows by applying the Cramér-Wold device.

□

C. Estimation for conditional variances. In this part, we detail the estimation of the conditional variances $\mathrm{Var}_P(\phi_P(X))$ and $\mathrm{Var}_P(\phi(X,Y,T) - \mathbb{E}_P[\phi(X,Y,T)\,|\,X])$ in the construction of our distribution shift measures, which is omitted from Appendix 2.C.

Recall that P is the underlying distribution of the "source" site, and Q is that of the "target" site. We write the influence function in the most general from $\phi(X,Y,T)$, though in the datasets it is a function of only (Y,T). We will use cross-fitting (**?**) with machine learning models for fitting the conditional mean functions.

The variance estimation only needs data from the source site, $\mathcal{D}_1 \overset{\text{i.i.d.}}{\sim} P$. We randomly split $\mathcal{D}_1$ into two folds $\mathcal{D}_1^{(1)} \cup \mathcal{D}_1^{(2)}$. For $k = 1,2$, we use $\mathcal{D}_1^{(k)}$ to fit the conditional mean function $\hat{\phi}^{(k)}(\cdot)$ for $\phi_P(\cdot) := \mathbb{E}_P[\phi(X,Y,T)\,|\,X = \cdot]$. Then, writing $\hat{\varphi}(X_i) = \hat{\varphi}^{(k)}(X_i)$ for $i \in \mathcal{D}_1 \backslash \mathcal{D}_1^{(k)}$, and $\hat{\varphi}(X_j) = \hat{\varphi}^{(k)}(X_j)$ for $j \in \mathcal{D}_2 \backslash \mathcal{D}_2^{(k)}$, we let

$$\hat{s}_{Y|X}^2 = \frac{1}{n_1}\sum_{i\in\mathcal{D}_1}\left(\phi(X_i,Y_i,T_i) - \hat{\varphi}(X_i)\right)^2,$$

$$\hat{s}_X^2 = \frac{1}{n_1}\sum_{i\in\mathcal{D}_1}\hat{\varphi}(X_i)\cdot\big(2\phi(X_i,Y_i,T_i)-\hat{\varphi}(X_i)\big)-\bigg(\frac{1}{n_1}\sum_{i\in\mathcal{D}_1}\phi(X_i,Y_i,T_i)\bigg)^2.$$

We define $\sigma(x) = \mathrm{Var}_P(\phi(X,Y,T)\,|\,X=x)$. Our estimators converge in $n^{-1/2}$ rate under standard slow convergence rates of nuisance components.

Theorem 1. *Suppose $\|\hat{\varphi}^{(k)}-\varphi\|_{L_2(P)} = o_P(n^{-1/4})$, and $\|\sigma\cdot(\hat{\varphi}^{(k)}-\varphi)\|_{L_2(P)} = o_P(1)$ for $k=1,2$. Then,*

$$\begin{pmatrix}\hat{s}_{Y|X}\\ \hat{s}_X\end{pmatrix} = \begin{pmatrix}s_{Y|X}\\ s_X\end{pmatrix} + \frac{1}{n_1}\sum_{i\in\mathcal{D}_1}\psi_1(X_i,Y_i,T_i) + o_P(1/\sqrt{n_1})$$

for some fixed function ψ with mean zero. As a result, each element is consistent and asymptotically $\sqrt{n}$-normal, and the asymptotic variances can all be consistently estimated.

Proof of Theorem 1. For simplicity, we write $D_i = (X_i,Y_i,T_i)$. By definition,

$$\begin{aligned}\hat{s}_{Y|X}^2 &= \frac{1}{n_1}\sum_{i\in\mathcal{D}_1}\big\{\phi(D_i)-\hat{\varphi}(X_i)\big\}^2\\ &= \frac{1}{n_1}\sum_{i\in\mathcal{D}_1}\big\{\phi(D_i)-\varphi(X_i))\big\}^2 + \frac{1}{n_1}\sum_{i\in\mathcal{D}_1}(\hat{\varphi}(X_i)-\varphi(X_i))^2 - \frac{2}{n_1}\sum_{i\in\mathcal{D}_1}(\hat{\varphi}(X_i)-\varphi(X_i))\cdot(\phi(D_i)-\varphi(X_i)).\end{aligned}$$

Since $\|\hat{\varphi}^{(k)}-\varphi\|_{L_2(P)} = o_P(n^{-1/4})$, and by Markov's inequality, we know that for any fixed $\epsilon>0$,

$$\begin{aligned}&\mathbb{P}\Bigg[\Bigg|\frac{1}{n_1/2}\sum_{i\notin\mathcal{D}_1^{(k)}}(\hat{\varphi}(X_i)-\varphi(X_i))^2\Bigg| > \epsilon\,\Bigg|\,\mathcal{D}_1^{(k)}\Bigg]\\ &\le \frac{2}{\epsilon^2}\mathbb{E}\Big[(\hat{\varphi}(X_i)-\varphi(X_i))^2\,\Big|\,\mathcal{D}_1^{(k)}\Big] = 2\|\hat{\varphi}^{(k)}-\varphi\|_{L_2(P)}^2/\epsilon^2 = o_P(n^{-1/2}).\end{aligned}$$

This implies

$$\frac{1}{n_1}\sum_{i\in\mathcal{D}_1}(\hat{\varphi}(X_i)-\varphi(X_i))^2 = o_P(1/\sqrt{n}).$$

Also, conditional on $\mathcal{D}_1^{(k)}$, note that $(\hat{\varphi}(X_i)-\varphi(X_i))\cdot(\phi(D_i)-\varphi(X_i))$ is i.i.d. with mean zero for all $i\notin\mathcal{D}_1^{(k)}$. Thus, by Markov's inequality, we have

$$\begin{aligned}&\mathbb{P}\Bigg[\Bigg|\frac{1}{n_1/2}\sum_{i\notin\mathcal{D}_1^{(k)}}(\hat{\varphi}(X_i)-\varphi(X_i))\cdot(\phi(D_i)-\varphi(X_i))\Bigg| > \epsilon\,\Bigg|\,\mathcal{D}_1^{(k)}\Bigg]\\ &\le \frac{1}{\epsilon^2}\mathbb{E}\Bigg[\Bigg(\frac{1}{n_1/2}\sum_{i\notin\mathcal{D}_1^{(k)}}(\hat{\varphi}(X_i)-\varphi(X_i))\cdot(\phi(D_i)-\varphi(X_i))\Bigg)^2\,\Bigg|\,\mathcal{D}_1^{(k)}\Bigg]\\ &= \frac{4}{\epsilon^2 n_1^2}\sum_{i\notin\mathcal{D}_i^{(k)}}\mathbb{E}\Big[(\hat{\varphi}(X_i)-\varphi(X_i))\cdot(\phi(D_i)-\varphi(X_i))^2\,\Big|\,\mathcal{D}_1^{(k)}\Big]\\ &= \frac{4}{\epsilon^2 n_1}\|\sigma\cdot(\hat{\varphi}^{(k)}-\varphi)\|_{L_2(P)}^2.\end{aligned}$$

Given that $\|\sigma\cdot(\hat{\varphi}^{(k)}-\varphi)\|_{L_2(P)} = o_P(1)$, we know

$$\frac{1}{n_1}\sum_{i\in\mathcal{D}_1}(\hat{\varphi}(X_i)-\varphi(X_i))\cdot(\phi(D_i)-\varphi(X_i)) = o_P(1/\sqrt{n}).$$

This means

$$\hat{s}_{Y|X}^2 - s_{Y|X}^2 = \frac{1}{n_1}\sum_{i\in\mathcal{D}_1}\big\{(\phi(D_i)-\varphi(X_i))^2 - s_{Y|X}^2\big\} + o_P(1/\sqrt{n}).$$

Similarly, by definition, and due to the fact that $\frac{1}{n_1}\sum_{i\in\mathcal{D}_1}\phi(D_i)-\mathbb{E}_P[\phi]=O_P(1/\sqrt{n})$,

$$\begin{aligned}
\hat{s}_X^2 &= \frac{1}{n_1}\sum_{i\in\mathcal{D}_1}\hat{\varphi}(X_i)(2\phi(D_i)-\hat{\varphi}(X_i)) - \Big(\frac{1}{n_1}\sum_{i\in\mathcal{D}_1}\phi(D_i)\Big)^2 \\
&= \frac{1}{n_1}\sum_{i\in\mathcal{D}_1}\varphi(X_i)(2\phi(D_i)-\varphi(X_i)) + \frac{2}{n_1}\sum_{i\in\mathcal{D}_1}(\hat{\varphi}(X_i)-\varphi(X_i))(\phi(D_i)-\varphi(X_i)) \\
&\quad - \frac{1}{n_1}\sum_{i\in\mathcal{D}_1}(\hat{\varphi}(X_i)-\varphi(X_i))^2 - \Big(\frac{1}{n_1}\sum_{i\in\mathcal{D}_1}\phi(D_i)\Big)^2 \\
&= \frac{1}{n_1}\sum_{i\in\mathcal{D}_1}\varphi(X_i)(2\phi(D_i)-\varphi(X_i)) - \Big(\frac{1}{n_1}\sum_{i\in\mathcal{D}_1}\phi(D_i)\Big)^2 + o_P(1/\sqrt{n}) \\
&= \frac{1}{n_1}\sum_{i\in\mathcal{D}_1}\varphi(X_i)(2\phi(D_i)-\varphi(X_i)) - (\mathbb{E}_P[\phi])^2 - 2\mathbb{E}_P[\phi]\Big(\frac{1}{n_1}\sum_{i\in\mathcal{D}_1}\phi(D_i)-\mathbb{E}_P[\phi]\Big) + o_P(1/\sqrt{n}) \\
&= \frac{1}{n_1}\sum_{i\in\mathcal{D}_1}\Big\{\varphi(X_i)(2\phi(D_i)-\varphi(X_i)) - 2\mathbb{E}_P[\phi]\phi(D_i) + (\mathbb{E}_P[\phi])^2\Big\} + o_P(1/\sqrt{n}),
\end{aligned}$$

which further implies

$$\hat{s}_X^2 - s_X^2 = \frac{1}{n_1}\sum_{i\in\mathcal{D}_1}\Big\{\varphi(X_i)(2\phi(D_i)-\varphi(X_i)) - \mathbb{E}_P[\phi^2] - 2\mathbb{E}_P[\phi](\phi(D_i)-\mathbb{E}_P[\phi])\Big\} + o_P(1/\sqrt{n}).$$

By Delta method, the above two results imply

$$\begin{aligned}
\hat{s}_{Y|X} - s_{Y|X} &= \frac{1}{n_1}\sum_{i\in\mathcal{D}_1}\frac{1}{2s_{Y|X}}\Big\{(\phi(D_i)-\varphi(X_i))^2 - s_{Y|X}^2\Big\} + o_P(1/\sqrt{n}), \\
\hat{s}_X - s_X &= \frac{1}{n_1}\sum_{i\in\mathcal{D}_1}\frac{1}{2s_X}\Big\{\varphi(X_i)(2\phi(D_i)-\varphi(X_i)) - \mathbb{E}_P[\phi^2] - 2\mathbb{E}_P[\phi](\phi(D_i)-\mathbb{E}_P[\phi])\Big\} + o_P(1/\sqrt{n}).
\end{aligned}$$

These give us the desired asymptotic expansion of the resulting estimators, with

$$\psi(X_i,Y_i,T_i) = \begin{pmatrix} \frac{1}{2s_{Y|X}}\Big\{(\phi(D_i)-\varphi(X_i))^2 - s_{Y|X}^2\Big\} \\ \frac{1}{2s_X}\Big\{\varphi(X_i)(2\phi(D_i)-\varphi(X_i)) - \mathbb{E}_P[\phi^2] - 2\mathbb{E}_P[\phi](\phi(D_i)-\mathbb{E}_P[\phi])\Big\} \end{pmatrix}.$$

We thus conclude the proof of Theorem 1. □
